# Involvement of Protein Kinase C in the Suppression of Apoptosis and in Polarity Establishment in *Aspergillus nidulans* under Conditions of Heat Stress

**DOI:** 10.1371/journal.pone.0050503

**Published:** 2012-11-28

**Authors:** Takuya Katayama, Hirotaka Uchida, Akinori Ohta, Hiroyuki Horiuchi

**Affiliations:** Department of Biotechnology, The University of Tokyo, Tokyo, Japan; University of Wisconsin - Madison, United States of America

## Abstract

The *pkcA* gene, which encodes a protein kinase C (PKC) in the filamentous fungus *Aspergillus nidulans*, is essential for its viability. However, little is known about its functions. To address this issue, we constructed and characterized temperature-sensitive mutants of *pkcA*. The conidia of these mutants swelled slightly and exhibited apoptotic phenotypes at 42°C. The apoptotic phenotypes were suppressed by an osmotic stabilizer. Under these conditions, the conidia swelled extensively and did not form germ tubes. Moreover, polarized distribution of F-actin was not observed. We then utilized deletion mutants of *bckA*, an ortholog of *Saccharomyces cerevisiae bck1* that encodes a mitogen-activated protein (MAP) kinase kinase kinase and functions downstream of PKC in the cell wall integrity pathway. These mutants exhibited apoptotic phenotypes at 42°C, but they did not show defects in polarity establishment under osmotically stabilized conditions. These results suggest that PkcA plays multiple roles during germination under conditions of heat stress. The first of these roles is the suppression of apoptosis induction, while the other involves polarity establishment. The former depends on the MAP kinase cascade, whereas the latter does not. In addition, repolarization, which was observed after depolarization in the wild-type strain and the *bckA* deletion mutant under conditions of heat stress, was not observed in the *pkcA*-ts mutant. This suggests that PkcA also plays role in polarity establishment during hyphal growth independent of the MAP kinase cascade under these conditions.

## Introduction

Protein kinase C (PKC) is a serine/threonine kinase that is conserved among eukaryotes and is of central importance in various signal-transduction processes. Although mammalian cells have at least 10 PKC isoforms, fungi have only 1 or 2 [1]. The budding yeast *Saccharomyces cerevisiae* has only 1 PKC-encoding gene, i.e., *pkc1*. Deletion of this gene is lethal under normal conditions; however, this lethality is suppressed by osmotic stabilization. Previous reports suggested that Pkc1p is involved in various cellular processes (reviewed in Levin, 2005 and 2011) [2], [3]. One of the main functions of Pkc1p is to activate the cell wall integrity (CWI) pathway. When cells are exposed to cell wall stress, Pkc1p is activated and phosphorylates Bck1p, resulting in the activation of a mitogen-activated protein (MAP) kinase cascade. This MAP kinase cascade includes Bck1p, Mkk1p and Mkk2p, and Slt2p, as a MAPKKK, MAPKKs, and a MAPK, respectively. Slt2p phosphorylates and activates a transcription factor, Rlm1p, which regulates the expression of many genes whose products are involved in cell wall biosynthesis [4]. In addition, Pkc1p is suggested to have other functions in polarity establishment, cell-cycle control, regulation of oligosaccharyl transferase, and control of chitin synthase distribution [2], [3]. However, the functions of Pkc1p at the molecular level are not well known.

PKCs have been identified and characterized in some filamentous fungi, with their structural features closely resembling those of yeasts, suggesting that PKCs of filamentous fungi have functions similar to those of yeasts [5]–[8]. PKCs are suggested to play a role in the CWI pathway of filamentous fungi as well as in yeasts [9], [10]. However, they also play roles in other cellular process. In *Neurospora crassa* and *Cochliobolus heterostrophus*, PKC is suggested to be essential for viability [6], [11]. In *N. crassa*, PKC is involved in a light-signaling pathway [7], [11] and it is activated by diacylglycerol and phorbol ester. It localizes at the hyphal tips, branch tips, and forming septa [12]. In *Aspergillus oryzae*, PKC is involved in the regulation of multimerization and localization of a Woronin body protein [13].

In *A. nidulans*, a single PKC-encoding gene, *pkcA*, was isolated and characterized. *pkcA* is essential for its viability even under conditions supplemented with osmotic stabilizers [14]–[17]. Repression of *pkcA* expression led to hypersensitivity to cell wall-perturbing agents and defects in the cell wall structure [15], [16]. In addition, *A. nidulans* has orthologs of the components of the CWI pathway [18]. These facts suggest that a CWI pathway similar to yeasts and other filamentous fungi is also present in *A. nidulans*, and that PkcA is involved in it. It was shown that PkcA localized to the hyphal apices, forming septa, and tips of phialides [16]. In addition, PkcA is suggested to function in conidiation, germination, nuclear division, secondary metabolism, unfolded-protein response, and farnesol-induced cell death [14]–[16], [19], [20].

In this study, to investigate PkcA function, we constructed and characterized *pkcA* temperature-sensitive (*pkcA*-ts) mutants. Our results suggest that PkcA is involved in the suppression of apoptosis induction and in polarity establishment under conditions of heat stress.

## Results

### Construction and Characterization of *pkcA* Temperature-sensitive Mutants

**Figure 1:**
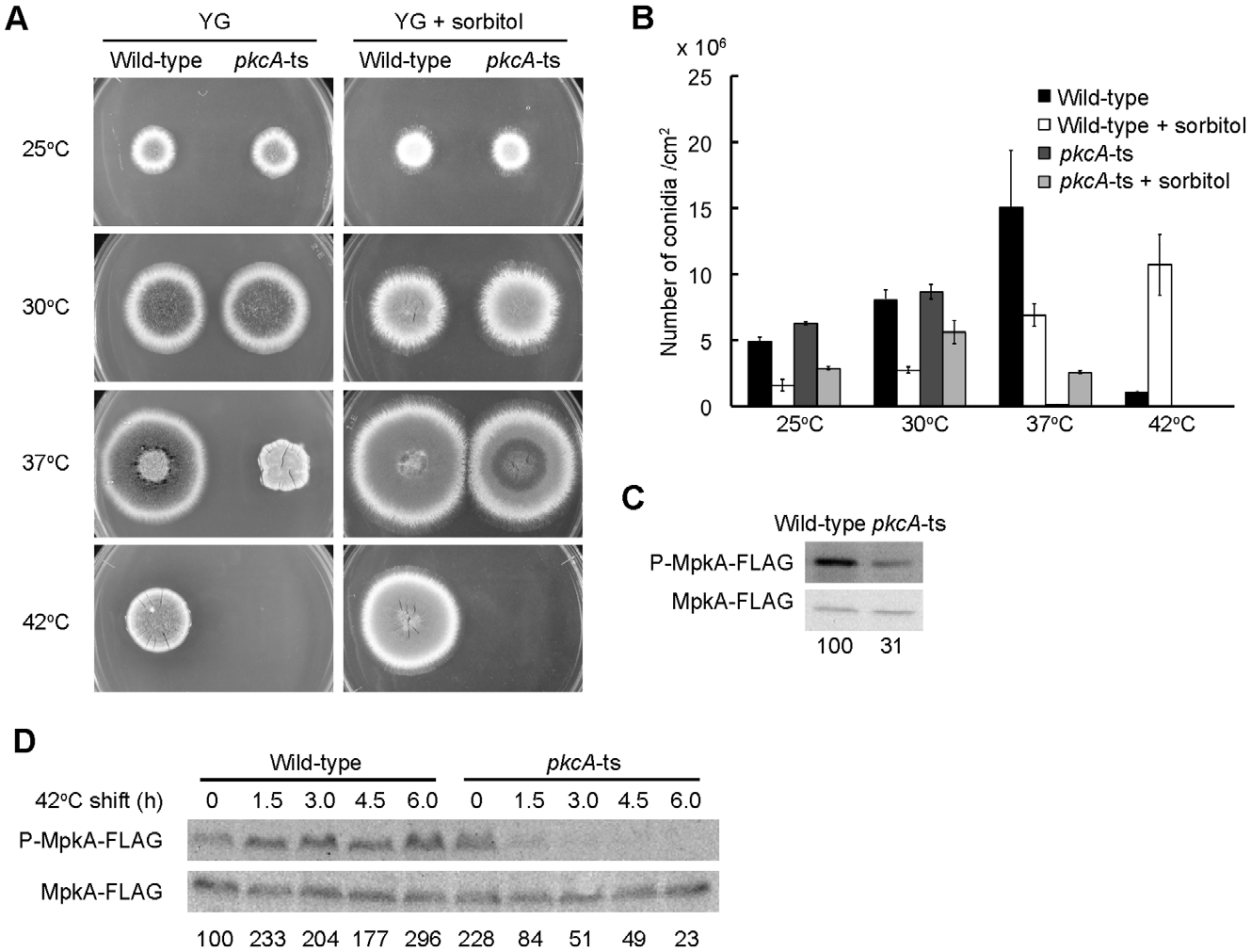
Characterization of the *pkcA*-ts mutant. A. Growth of the pkcA-ts-2 mutant. 10^3^ conidia from the A1149 strain (Wild-type) and the pkcA-ts-2 mutant (*pkcA*-ts) were inoculated on the YG plate and the YG plate supplemented with 1.2 M sorbitol, and incubated for 72 h at indicated temperatures. B. Conidiation efficiency of the pkcA-ts-2 mutant. Conidia from the A1149 strain (Wild-type) and the pkcA-ts-2 mutant (*pkcA*-ts) were inoculated as described above and the number of conidia was measured. Data are shown as means ± S.E.M. of three independent experiments. C. Phosphorylation levels of MpkA-FLAG at 37°C. Cell extracts were prepared from A1149/MF-1 (Wild-type) and pkcA-ts/MF-1 (*pkcA*-ts), which were incubated on the YG plate for 16 h at 37°C. The upper panel indicates the phospholyrated MpkA-FLAG (P-MpkA-FLAG). The lower panel indicates the MpkA-FLAG (MpkA-FLAG). The numbers under the lower panel indicate the ratio of MpkA-FLAG phosphorylation comparing to that in the wild-type strain. D. Phosphorylation levels of MpkA-FLAG at 42°C. Cell extracts were prepared from A1149/MF-1 (Wild-type) and pkcA-ts/MF-1 (*pkcA*-ts), which were incubated on the MMG plate for 20 h at 30°C, shifted to 42°C and incubated for indicated time periods at 42°C. The upper panel indicates the phospholyrated MpkA-FLAG (P-MpkA-FLAG). The lower panel indicates the MpkA-FLAG (MpkA-FLAG). The numbers under the lower panel indicate the ratio of MpkA-FLAG phosphorylation at the indicated time periods comparing to that in the wild-type strain at 0 h.

To investigate the function of PkcA, we constructed conditional *pkcA* mutants. In *S. cerevisiae,* several conditional *pkc1* mutants have been created, including a strain carrying the *pkc1–2* allele, which is temperature-sensitive for its growth. The *pkc1–2* allele carries a C to T transition at nucleotide position 3068, which results in the replacement of Pro1023 with Leu [21]. Since Pro1023 of Pkc1p is conserved in the amino acid sequence of PkcA of *A. nidulans* (Pro959 of PkcA), we constructed pkcA-ts-2, −3, and −5 strains that produce mutated PkcA in which Pro959 was replaced by Leu; then, we characterized these strains (Table 1). Since all of them exhibited the same phenotypes under the conditions tested, we chose the pkcA-ts-2 mutant for further analysis. When grown on the YG plate for 72 h, the colony diameter of the mutant was almost the same as that of the wild-type strain (A1149) at 25°C and 30°C. In contrast, at 37°C, the colony diameter of the mutant was approximately 60% of that of the wild-type strain, and the mutant did not form colonies at 42°C (Fig. 1A). A previous study showed that the lethality of the *S. cerevisiae pkc1* deletion mutant is suppressed by an osmotic stabilizer at 30°C, and that the temperature sensitivity of the *pkc1–2* mutant is partially suppressed by CaCl_2_ [21]. The pkcA-ts-2 mutant did not form colonies on the YG plate supplemented with 1.2 M sorbitol as an osmotic stabilizer or 200 mM CaCl_2_ at 42°C (Fig. 1A and data not shown). The pkcA-ts-2 mutant formed nearly the same number of conidia as the wild-type strain at 25°C and 30°C. However, the conidiation efficiency of the mutant was 0.14 conidia/cm^2^ at 37°C, which was 100-fold fewer than that of the wild-type strain. Under the condition with the osmotic stabilizer 1.2 M sorbitol at 37°C, the conidiation efficiency of the wild-type strain was 6.90 conidia/cm^2^ and that of the mutant was 2.56 conidia/cm^2^, suggesting that the reduction of the conidiation efficiency of the mutant was partially suppressed by the osmotic stabilizer (Fig. 1B). The pkcA-ts-2 mutant lacks *nkuA*. It encodes the *A. nidulans* ortholog of mammalian KU70 that is involved in non-homologous end-joining. To avoid the effect of *nkuA* deletion on the phenotypes of the pkcA-ts-2 mutant, we constructed *pkcA*-ts mutants with *nkuA* ^+^ alleles, namely, pkcA-ts nkuA^+^-1 and pkcA-ts nkuA^+^-2 (see Materials and Methods). These mutants showed almost the same phenotypes as the pkcA-ts-2 mutant, suggesting that the deletion of *nkuA* does not affect the phenotypes of the *pkcA*-ts mutants.

**Table 1:** *A. nidulans* strains used in this study.

Strain	Genotype	Source
FGSC A26	*biA1*	FGSC^a^
FGSC A1149	*pyrG89 pyroA4 nkuA*::*argB*	FGSC^a^
FGSC A1145	*pyrG89 pyroA4 riboB2 nkuA*::*argB*	FGSC^a^
pkcA-ts-2,3,5	*pyrG89 pyroA4 riboB2 nkuA*::*argB pkcA*::*pkcA*(P959L)-*riboB*	This study
A1149LA-1,2	*pyrG89*::*alcA*(p)-*lifeact-EGFP-pyrG pyroA4 nkuA*::*argB*	This study
pkcA-ts-LA-1,2	*pyrG89*::*alcA*(p)*-lifeact-EGFP-pyrG pyroA4 riboB2 nkuA*::*argB*	This study
	*pkcA*::*pkcA*(P959L)*-riboB*	
ΔbckA-1.2	*pyrG89 pyroA4* Δ*bckA*::*pyroA nkuA*::*argB*	This study
ΔbckALA-1,2	*pyrG89*::*alcA*(p)-*lifeact*-*EGFP*-*pyrG pyroA4* Δ*bckA*::*pyroA*; nkuA::argB	This study
A1149/MF-1,2	*pyrG89 pyroA4 nkuA*::*argB mpkA*::*mpkA-*3x*FLAG-pyroA*	This study
pkcA-ts/MF-1,2	*pyrG89 pyroA4 riboB2 nkuA*::*argB pkcA*::*pkcA*(P959L)-*riboB*	This study
	*mpkA*::*mpkA*-3x*FLAG-pyroA*	
alcA(p)-pkcA-3,4	*pyrG89 pyroA4 riboB2 nkuA*::*argB pkcA*::*riboB*-*alcA*(p)-*pkcA*	This study
ΔmpkA-1,2,8	*pyrG89 pyroA4 nkuA*::*argB* Δ*mpkA*::*pyrG*	This study
A1149/pyroA	*pyrG89 pyroA4 nkuA*::*argB* [pUCpyroA]	Yamazaki *et al.* unpublished
A1149/pyrG-1, 2, 8	*pyrG89 pyroA4 nkuA*::*argB* [ppyrG+750]	This study
ABPU1	*biA1 pyrG89 wA3 argB2 pyroA4*	[43]
pkcA-ts nkuA^+^	*pyrG89 wA3 argB2 pyroA4 pkcA*::*pkcA*(P959L)-*riboB*	This study (ABPU1 × pkcA-ts-2)
ΔpkcA-h1, 2, 3	Heterokaryon	This study
	*pyrG89/pyrG89 pyroA4*/*pyroA4 nkuA*::*argB*/*nkuA*::*argB*	
	*riboB2*/*riboB2 pkcA*/Δ*pkcA*::*riboB*	

a Fungal Genetics Stock Center, Kansas City, MO, USA.

In *S. cerevisiae*, Pkc1p activity is measured by detecting the phosphorylation level of Slt2p, which is a MAP kinase that functions downstream of Pkc1p [22]. To measure PkcA activity, we constructed a *pkcA*-ts mutant (pkcA-ts/MF-1) and a relevant wild-type strain (A1149/MF-1) that produced the Slt2p ortholog of *A. nidulans*, i.e., MpkA, tagged with 3 repeats of a FLAG epitope at its C-terminus (MpkA-FLAG). The phosphorylation level of MpkA-FLAG in the *pkcA*-ts mutant incubated on the YG medium at 37°C was lower than that of the wild-type strain (Fig. 1C). The *pkcA*-ts mutant on the MMG medium at 30°C was shifted to 42°C, and the phosphorylation level of MpkA-FLAG was measured. In the wild-type strain, the phosphorylation level of MpkA-FLAG increased at 1.5 h after the temperature shift and did not significantly change until 6 h. In contrast, the phosphorylation level of MpkA-FLAG in the *pkcA*-ts mutant rapidly decreased after the shift to 42°C (Fig. 1D). The phosphorylation level of MpkA-FLAG in the mutant was higher than that in the wild-type strain at 0 h, suggesting that the PkcA activity of the mutant is higher than that of the wild-type strain at 30°C. The mutated PkcA with Leu959 may exhibit slight defects at 30°C. When grown on the MMG plate supplemented with 1.2 M sorbitol, the phosphorylation level of MpkA-FLAG in the mutant was also higher than that in the wild-type strain at 30°C. The phosphorylation level in the mutant rapidly decreased after the shift to 42°C under this condition (Fig. S1).

### DNA Degradation Occurred in the *pkcA*-ts Mutant Incubated at 42°C

**Figure 2:**
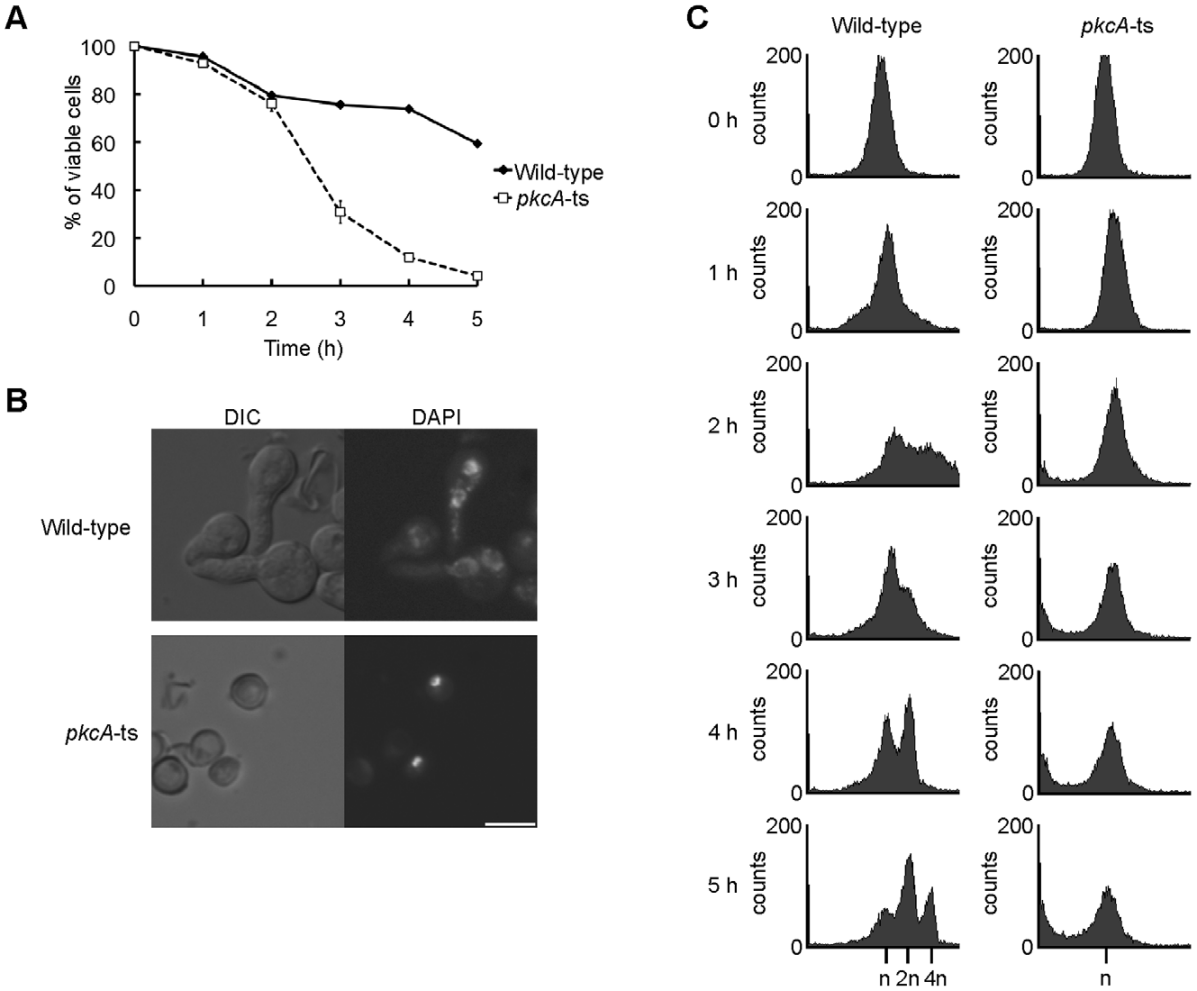
DNA degradation occurred in the *pkcA*-ts mutant at 42°C during germination. A. The viabilities of the conidia of the A1149 strain (Wild-type) and the pkcA-ts-2 mutant (*pkcA*-ts) incubated in the YG medium for indicated time periods at 42°C. Data are shown as means ± S.E.M, of three independent experiments. B. Conidia from the A1149 strain (Wild-type) and the pkcA-ts-2 mutant (*pkcA*-ts) were incubated in the YG medium for 5 h at 42°C, and then fixed and stained with DAPI. Bar, 5 µm. C. Conidia from the A1149 strain (Wild-type) and the pkcA-ts-2 mutant (*pkcA*-ts) were incubated in the YG medium for indicated times at 42°C, and analyzed by flow cytometry. When the wild-type strain was incubated at 42°C, sharp peak was not observed at 2 h of incubation. The cause of this phenomenon is currently unclear.

The viability of the pkcA-ts-2 mutant was remarkably decreased when the cells were incubated in the YG medium at 42°C for 3h (Fig. 2A). This indicates that PkcA is required during the early steps of germination under conditions of heat stress. To investigate the terminal phenotype of the pkcA-ts-2 mutant during germination, we observed the morphologies of the germinating conidia of the mutant incubated for 5 h at 42°C. Although the conidia of the wild-type strain swelled and formed germ tubes, those of the mutant swelled incompletely and did not form germ tubes (Fig. 2B); some of them lysed. While the cells of the wild-type strain had 2 or 4 nuclei, all cells of the mutant had only 1 nucleus (Fig. 2B), indicating that nuclear division did not occur in the mutant at 42°C. Next, we measured the DNA contents of the mutant cells by flow cytometric analysis. In the wild-type strain, the number of cells containing replicated DNA (indicated as 2n and 4n in Fig. 2C) increased. In the mutant, it did not increase and the number of cells whose DNA content was less than that in the conidia at 0 h (indicated as n) gradually increased from 2 h to 5 h (Fig. 2C). These results suggest that when the mutant is incubated at 42°C, DNA replication does not proceed, DNA is degraded, and nuclear division does not occur.

### The *pkcA*-ts Mutant Exhibits Phenotypes Characteristic of Apoptosis at 42°C

**Figure 3:**
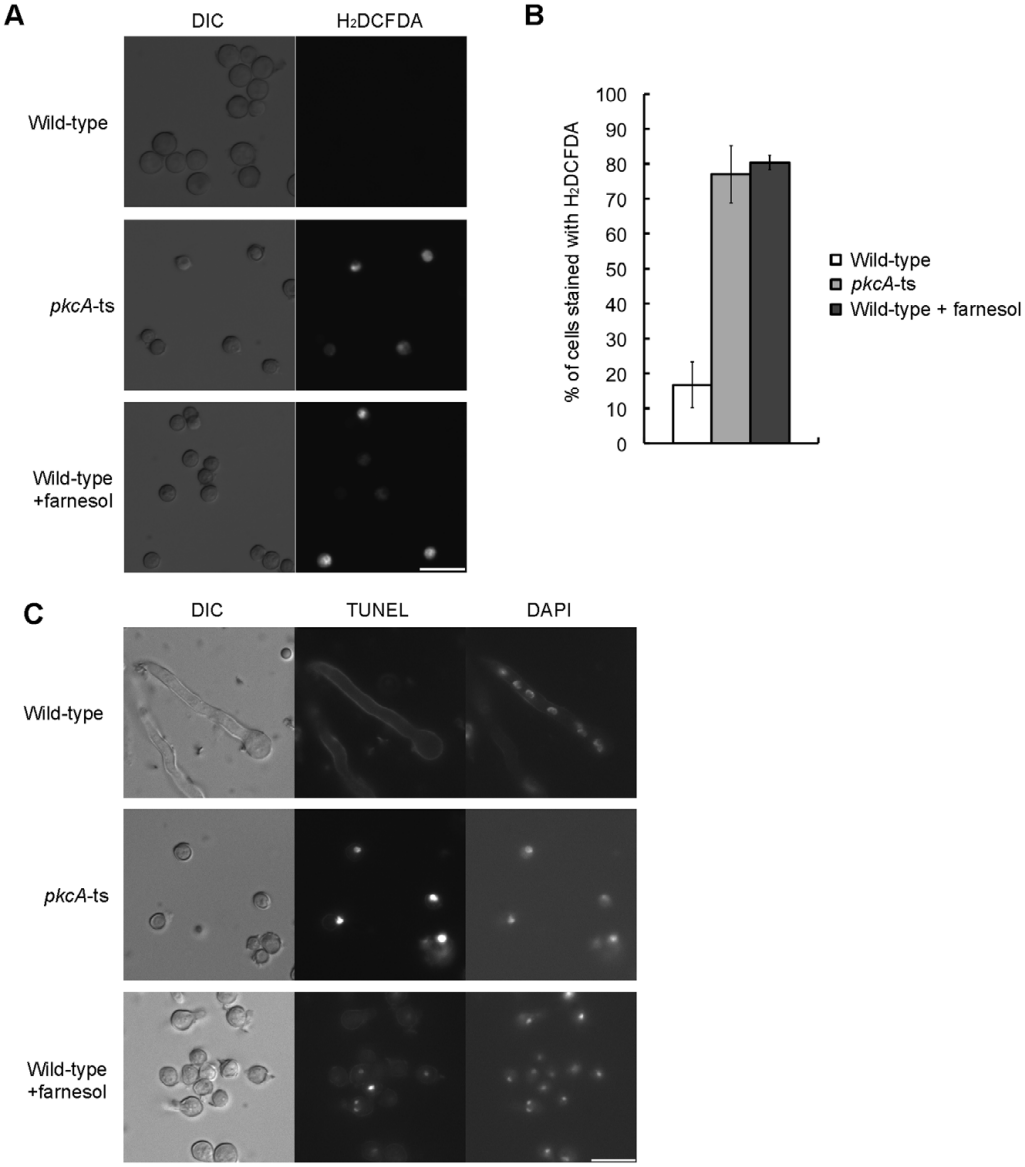
The *pkcA*-ts mutant exhibited apoptotic phenotypes at 42°C. A. Conidia from the A1149 strain (Wild-type) and the pkcA-ts-2 mutant (*pkcA*-ts) were incubated in the YG medium for 4 h at 42°C and treated with H_2_DCFDA. Conidia from the A1149 strain pretreated with farnesol were treated with H_2_DCFDA and indicated as control. Bar, 10 µm. B. The ratios of cells stained with H_2_DCFDA under the conditions of panel A. Data are shown as means ± S.E.M, of three independent experiments. C. Conidia from the A1149 strain (Wild-type) and the pkcA-ts-2 mutant (*pkcA*-ts) were inoculated in the liquid YG medium on coverslips, incubated for 8 h at 42°C and fixed, labeled with TUNEL and stained with DAPI. Conidia from the A1149 strain pretreated with farnesol were labeled with TUNEL and stained with DAPI, and indicated as control. Bar, 10 µm.

Since DNA degradation occurs when apoptosis is induced by certain treatment, we examined whether apoptosis was induced in the pkcA-ts-2 mutant at 42°C. When apoptosis is induced, specific phenotypes such as reactive oxygen species (ROS) accumulation and DNA fragmentation are observed in most eukaryotic cells [23]. We monitored ROS accumulation by using the oxidant-sensitive probe 2′,7′-dichlorodihydrofluorescein diacetate (H_2_DCFDA), which undergoes oxidization by ROS, and then, fluoresces. In addition, we monitored DNA fragmentation in the mutant conidia incubated in the YG medium at 42°C by the TUNEL assay. We used conidia treated with 100 µM farnesol as a positive control in these experiments, because it has been shown to induce apoptosis [24]. When the wild-type strain was incubated in the YG medium at 42°C, ROS-accumulating cells were less than 20% of total population and DNA fragmentation was hardly detected. In the case of the pkcA-ts-2 mutant, they were more than 70% and DNA fragmentation was detected (Fig. 3). These results strongly suggest that apoptosis is induced in the mutant at 42°C.

### BckA and MpkA Regulate Apoptosis Induction at 42°C

**Figure 4:**
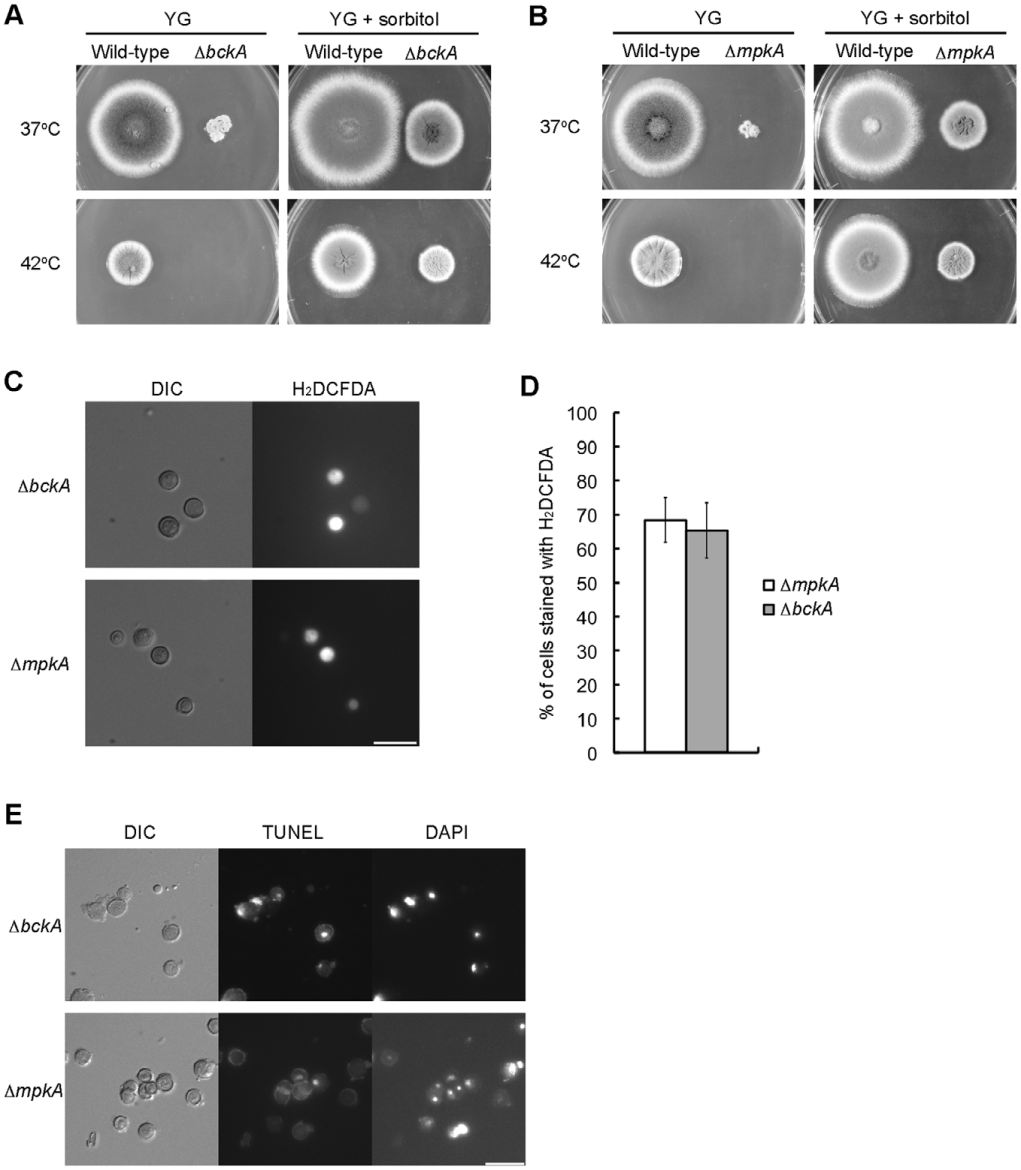
The *bckA* deletion mutant and the *mpkA* deletion mutant exhibited apoptotic phenotypes at 42°C. A. 10^3^ conidia from the A1149/pyroA strain (Wild-type) and the *bckA* deletion mutant (Δ*bckA*) were inoculated on the YG plate and the YG plate supplemented with 1.2 M sorbitol, and incubated for 72 h at indicated temperatures. B. 10^3^ conidia from the A1149/pyrG-1 strain (Wild-type) and the *mpkA* deletion mutant (Δ*mpkA*) were inoculated on the YG plate and the YG plate supplemented with 1.2 M sorbitol, and incubated for 72 h at indicated temperatures. C. Conidia from the *bckA* deletion mutant (Δ*bckA*) and the *mpkA* deletion mutant (Δ*mpkA*) were incubated in the YG medium for 4 h at 42°C, and then treated with H_2_DCFDA. Bar, 10 µm. D. The ratios of cells stained with H_2_DCFDA under the conditions of panel C. Data are shown as means ± S.E.M, of three independent experiments. E. Conidia from the *bckA* deletion mutant (Δ*bckA*) and the *mpkA* deletion mutant (Δ*mpkA*) were inoculated in liquid YG medium on coverslips, incubated for 8 h at 42°C and then fixed, labeled with TUNEL and stained with DAPI. Bar, 10 µm.

In *S. cerevisiae*, Pkc1p activates a MAP kinase cascade consisting of Bck1p, Mkk1/2p, and Slt2p. It is possible that their orthologs in *A. nidulans* are involved in the suppression of apoptosis induction. We constructed deletion mutants of *bckA,* a *BCK1* ortholog, or *mpkA*, an *SLT2* ortholog in *A. nidulans* (see Text S1). When the *bckA* deletion mutant and the *mpkA* deletion mutant were incubated on the YG plate, they formed smaller colonies than the wild-type strain and hardly formed any conidia at 37°C; they did not grow at 42°C (Fig. 4A and B). When the *bckA* deletion mutant and the *mpkA* deletion mutant were incubated on the YG plate supplemented with 1.2 M sorbitol, their defects in the growth and conidiation at 37°C were partially suppressed, and they formed colonies even at 42°C (Fig. 4A and B). We tested whether the apoptotic phenotypes were also observed in the *bckA* deletion mutant and the *mpkA* deletion mutant incubated at 42°C. In the both deletion mutants, the ratios of ROS-accumulating cells were more than 60% and DNA fragmentation was detected (Fig. 4C, D and E). These results suggest that BckA and MpkA are also involved in the suppression of apoptosis induction at 42°C.

### The Defective DNA Replication does not Induce Apoptosis at 42°C

Because DNA replication did not proceed in the germinating *pkcA*-ts mutant conida at 42°C, we explored possibility that the apoptosis observed in the mutant was induced by the defective DNA replication. When DNA synthesis of the wild-type conidia was inhibited by the treatment with 100 mM Hydroxyurea (HU) at 42°C, neither cell population of higher DNA content nor that of lower DNA content did not increase until 5 h. In the case of the *pkcA*-ts mutant, cell population with less DNA than 1n increased from 2 h (Fig. S2A). Furthermore, the HU-treated wild-type strain did not exhibit the apoptotic feature at 42°C, whereas in the HU-treated *pkcA*-ts mutant, ROS-accumulating cells were more than 60% of the total population and DNA fragmentation was detected in them (Fig. S2B, C, and D). These results suggest that defective DNA replication does not induce apoptosis at 42°C.

### Apoptosis is Not Induced in the *pkcA*-ts Mutant at 37°C

Since the phosphorylation level of MpkA-FLAG in the *pkcA*-ts mutant at 37°C was lower than that in the wild-type strain, we addressed whether apoptosis of the mutant is induced even at 37°C. Conidial *pkcA*-ts cell population containing replicated DNA increased after the start of incubation at 37°C, but cells containing less DNA than 1n did not increase (Fig. S3A). Futhermore, ROS-accumulating cell population was less than 20% and DNA fragmentation was hardly detected in the mutant as observed in the wild-type strain (Fig. S3B, C, and D). These results indicate that apoptosis is not induced in the mutant conidia at 37°C. Besides, neither cell wall stresses (by the treatment with calcofluor white, congo red, or micafungin) nor endoplasmic reticulum stress (i.e. dithiothreitol treatment) induced apoptosis in the *pkcA*-ts mutant at 37°C. Population of ROS-accumulating cells slightly increased in the mutant by the treatment of micafungin (Fig. S3E and F).

### PkcA and the MAP Kinase Cascade in the CWI Pathway are Involved in Farnesol Sensitivity

**Figure 5:**
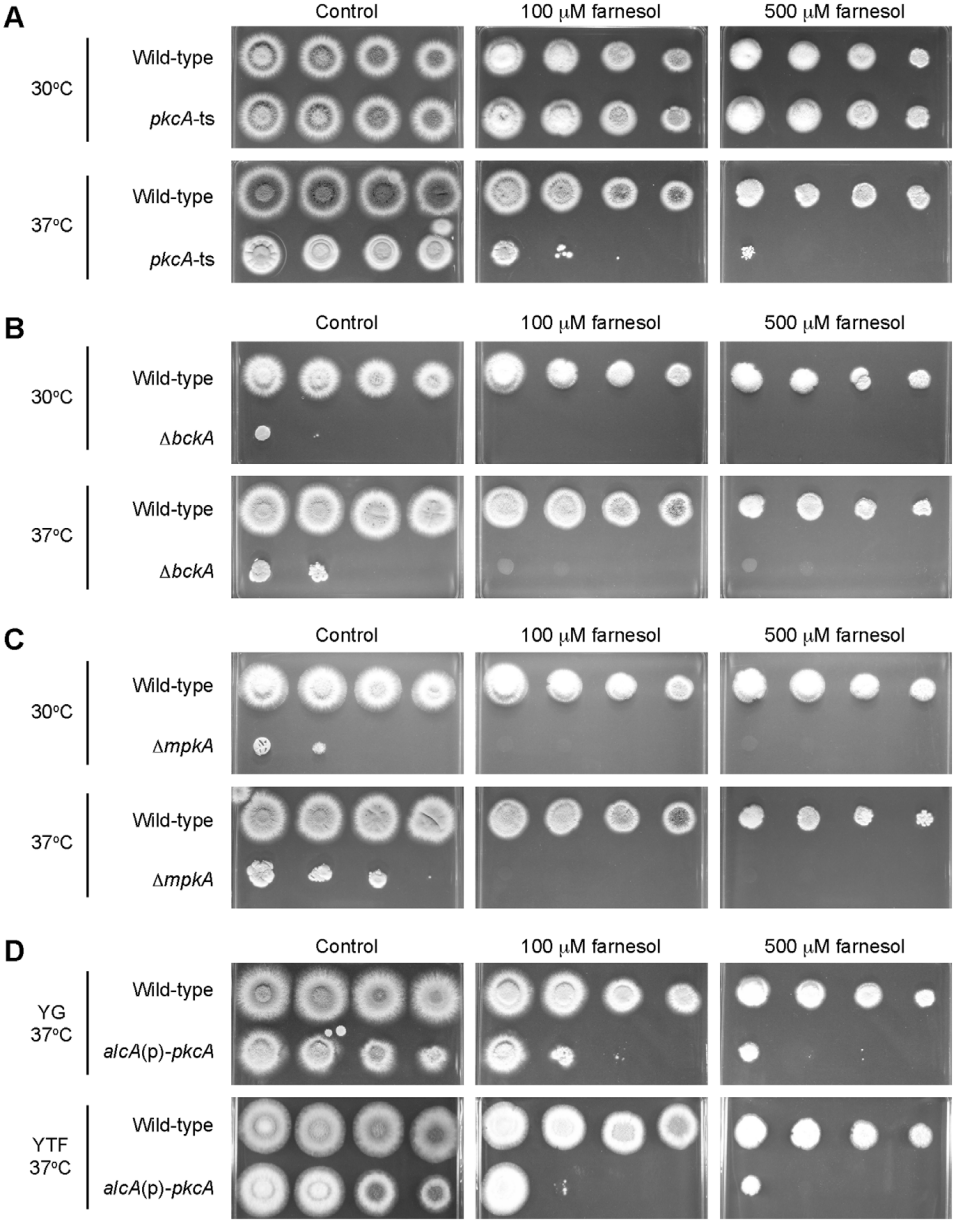
The sensitivity to farnesol of the pkcA-ts-2 mutant. Aliquots (5 ml) of 10-fold dilutions derived from a starting suspention of 1.0×10^8^ conidia ml^−1^ of the A1149 strain (wild-type) and the pkcA-ts-2 mutant (*pkcA*-ts) (A), the A1149/pyroA strain (wild-type) and the *bckA* deletion mutant (Δ*bckA*) (B), and the A1149/pyrG-1 strain (wild-type) and the *mpkA* deletion mutant (Δ*mpkA*) (C) were spotted on the YG plates supplemented with the 0, 0.1, 0.5 mM farnesol, and the A1149 strain (wild-type) the alcA(p)-pkcA-3 strain (*alcA*(p)-*pkcA*) (D) were spotted on the indicated plates supplemented with the 0, 0.1, 0.5 mM. The plates were incubated at the indicated temperature for 48 h.

Since the deletion mutants of *mkk2*, an *MKK1*/*2* ortholog, or *mpkA*, an *SLT2* ortholog, are sensitive to farnesol in *Aspergillus fumigatus* [25], it is possible that PKC and the MAP kinase cascade are also involved in the suppression of farnesol-induced apoptosis in *A. nidulans*. To investigate this possibility, we examined their sensitivities to farnesol of the pkcA-ts-2 mutant, the *bckA* deletion mutant, and the *mpkA* deletion mutant. We also examined the sensitivity of the alcA(p)-pkcA-3 strain in which *pkcA* was expressed under the control of an *alcA* promoter (*alcA*(p)). The pkcA-ts-2 mutant was hypersensitive to farnesol at 37°C, the temperature at which the phosphorylation level of MpkA in this mutant was lower than that in the wild-type strain (Fig. 5A). In addition, the *mpkA* deletion mutant and the *bckA* deletion mutant are also sensitive to farnesol (Fig. 5B and C). These results suggest that PKC and the MAP kinase cascade in the CWI pathway are involved in the suppression of farnesol-induced apoptosis in *A. nidulans*. The alcA(p)-pkcA-3 strain was hypersensitive to farnesol on the *alcA*(p)-inducing YTF medium (Fig. 5D).

### PkcA Plays Another Role During Germination under Conditions of Heat Stress

**Figure 6:**
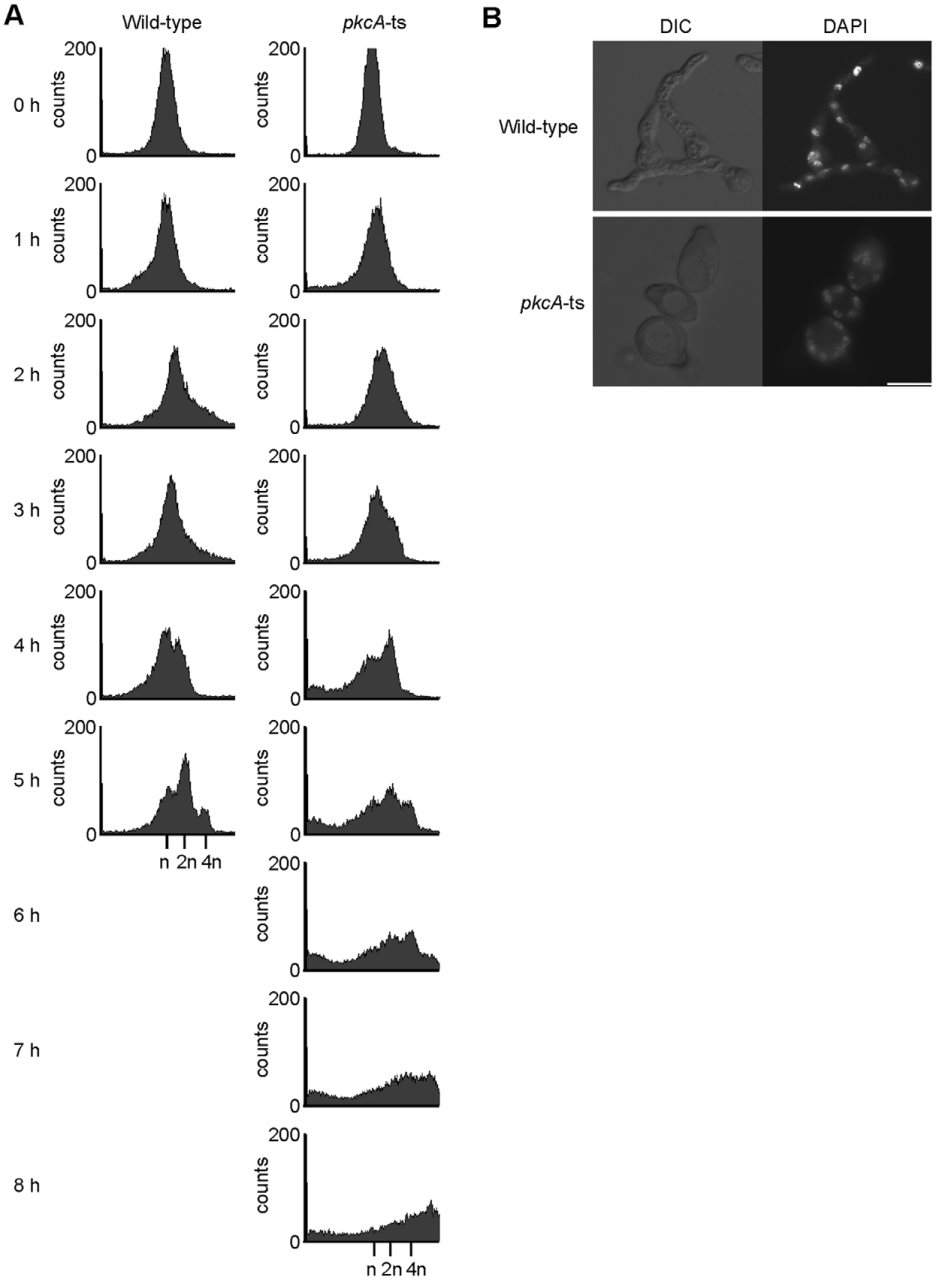
The osmotic stabilizer suppresses the DNA degradation of the *pkcA*-ts mutant at 42°C. A. Conidia from the A1149 strain (Wild-type) and the pkcA-ts-2 mutant (*pkcA*-ts) were incubated in the YG medium supplemented with 1.2 M sorbitol for indicated time periods at 42°C, and then analyzed by flow cytometry. B. Conidia from the A1149 strain (Wild-type) and the pkcA-ts-2 mutant (*pkcA*-ts) were incubated in the YG medium supplemented with 1.2 M sorbitol for 9 h at 42°C, and then fixed and stained with DAPI. Bar, 10 µm.

To determine whether an osmotic stabilizer suppressed apoptosis induction in the pkcA-ts-2 mutant, we monitored the DNA contents of the mutant cells grown with 1.2 M sorbitol at 42°C by flow cytometric analysis. In the case of the pkcA-ts-2 mutant, cells containing replicated DNA increased, while a small population of cells containing less DNA than 1n appeared at 4 h, but thereafter they did not much increase until 8 h (Fig. 6A). This result indicates that the osmotic stabilizer largely suppresses apoptosis induction in the mutant at 42°C. To investigate whether nuclear division occurs under this condition, we observed the nuclei of the pkcA-ts-2 mutant incubated with 1.2 M sorbitol for a longer period at 42°C. When incubated in the YG medium for 9 h at 42°C, most of the mutant cells swelled extensively and did not form germ tubes (Fig. 6B); some of them lysed before swelling. Multiple nuclei were observed in the extensively swollen cells (Fig. 6B). The average number of nuclei in the mutant from 3 independent experiments was the same as that in the wild-type strain (Fig. S4). These results indicate that nuclear division progressed normally in the pkcA-ts-2 mutant incubated under osmotically protective conditions. This observation suggests that the pkcA-ts-2 mutant has a defect in the germination process, especially in polarity establishment at 42°C.

### Lifeact as a Reporter to Detect F-actin Distribution in *A. nidulans*

**Figure 7:**
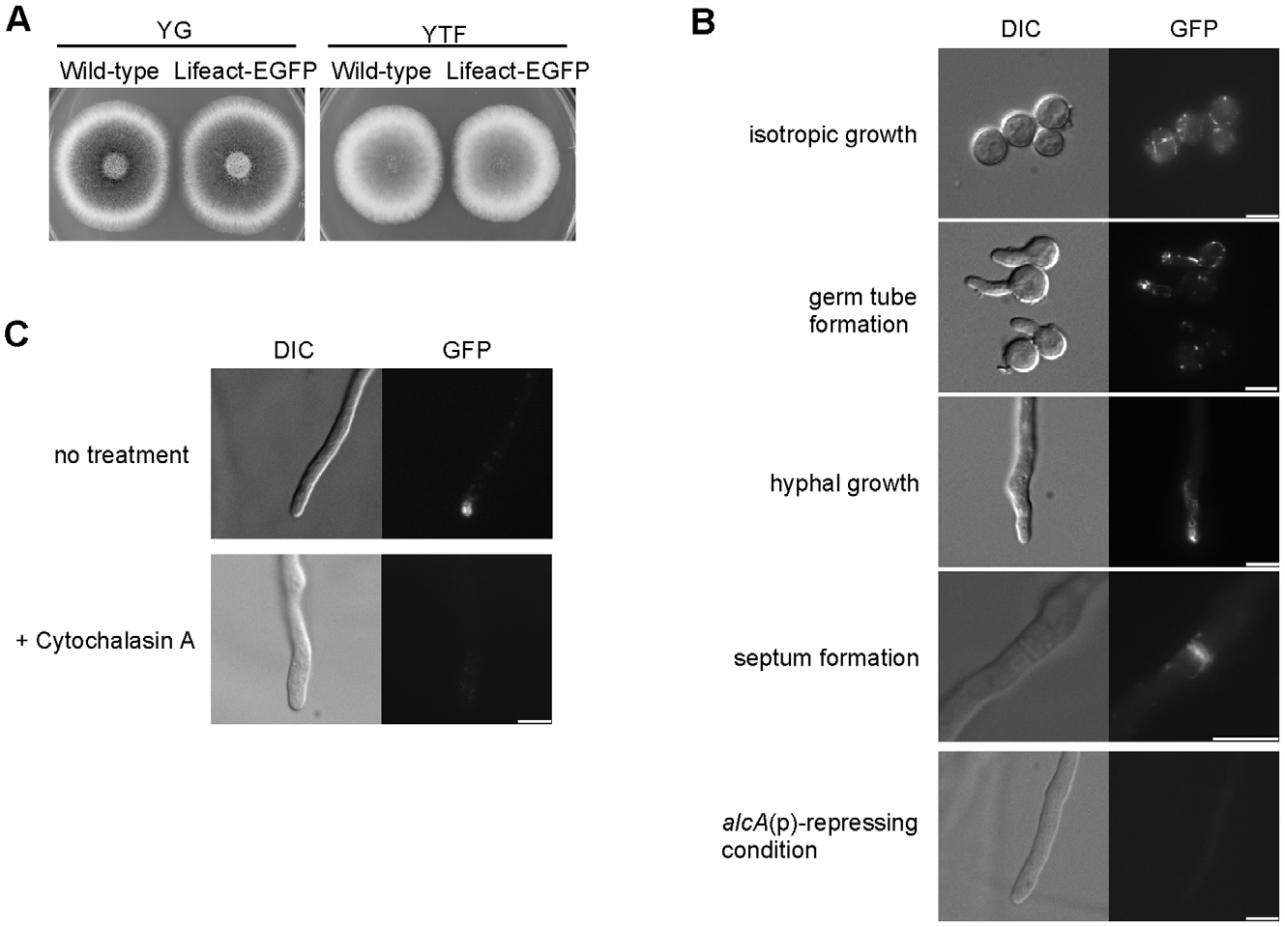
Lifeact-EGFP can be used as a reporter to detect F-actin in *A. nidulans*. A. 10^3^ conidia from the A1149/pyrG-1 strain (Wild-type) and the A1149LA-1 strain (Lifeact-EGFP) were inoculated under the *alcA*(p)-repressing condition (YG) and the *alcA*(p)-inducing condition (YTF), and incubated for 72 h at 37°C. B. Conidia from the A1149LA-1 strain (Lifeact-EGFP) were grown in the YT medium at 37°C for 4 h (isotropic growth) or for 5 h (germ tube formation), or incubated on coverslips submerged in the supplemented MMTF medium for 30 h (hyphal growth and septum formation) or in the supplemented MMG medium for 30 h (*alcA*(p)-repressing condition). Bar, 5 µm. C. Conidia of the A1149LA-1 strain (Lifeact-EGFP) were inoculated on coverslips and incubated in the MMTF medium for 16 h at 37°C, and treated with Cytochalasin A. Bar, 5 µm.

In various organisms, actin polymerization is required for polarity establishment, and the formation of actin filaments is a marker of the establishment of cell polarity. The first 17 amino acids of Abp140p of *S. cerevisiae* (Lifeact) can specifically bind to F-actin, and Lifeact fused with EGFP is an effective reporter to detect F-actin distribution in mammalian cells [26]. In *N. crassa*, Lifeact-EGFP has also been used as the reporter to study F-actin distribution [27], [28]. To investigate the function of PkcA in polarity establishment, we constructed a strain in which Lifeact-EGFP was expressed under the control of *alcA*(p) (A1149LA-1) (see Materials and Methods). This strain grew and formed conidia as well as the wild-type strain under the *alcA*(p)-inducing conditions (Fig. 7A), suggesting that the overproduction of Lifeact-EGFP did not affect its growth and differentiation. When the A1149LA-1 strain was incubated under the *alcA*(p)-repressing conditions, the fluorescence of Lifeact-EGFP was not detected (Fig. 7B). We observed the distribution of Lifeact-EGFP in the A1149LA-1 strain under the *alcA*(p)-inducing conditions. The YT medium was used to observe the distribution of Lifeact-EGFP during germination to avoid the repression of *alcA*(p) by fructose, while during hyphal growth, we used the MMTF medium to reduce background fluorescence. During isotropic growth, the fluorescence of Lifeact-EGFP was observed as punctate and filamentous structures in the cytoplasm (Fig. 7B). During the formation of germ tubes and hyphal growth, Lifeact-EGFP was observed as spots at the cortex of the tips of the germ tubes and hyphae, and as filamentous structures in the cytoplasm (Fig. 7B). Lifeact-EGFP was also observed at forming septa (Fig. 7B). The distribution patterns of Lifeact-EGFP were similar to those observed by indirect immunofluorescence microscopy using an anti-actin antibody [29]. When the hyphae were treated with cytochalasin A, which prevents actin polymerization, the fluorescence of Lifeact-EGFP disappeared (Fig. 7C). These observations suggest that Lifeact-EGFP is an effective reporter to detect F-actin distribution in *A. nidulans*.

### PkcA is Required for Polarity Establishment during Germination

**Figure 8:**
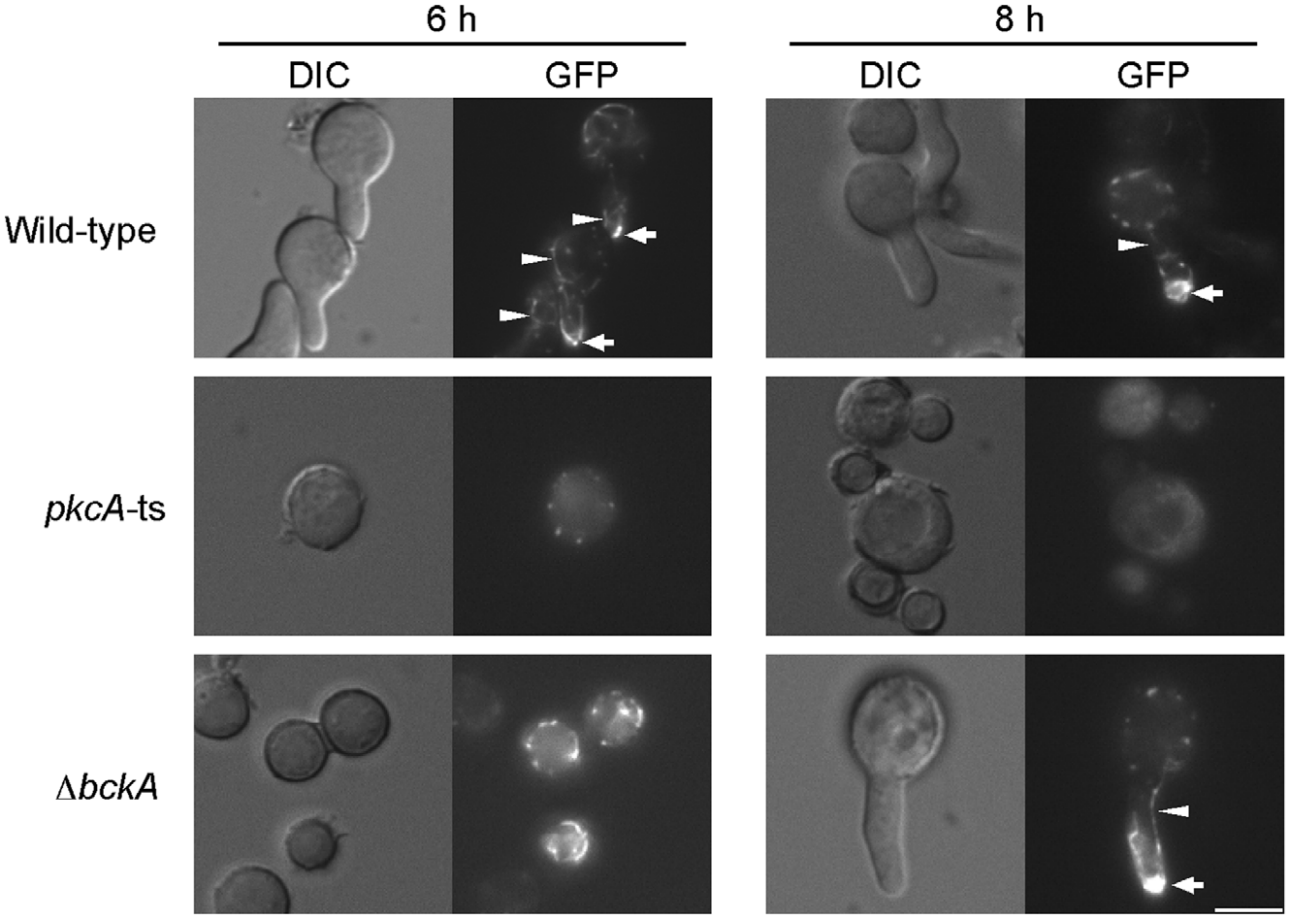
The *pkcA*-ts mutant exhibited the defect in a polarity establishment when grown with an osmotic stabilizer at 42°C. Conidia from the A1149LA-1 strain (Wild-type), the pkcA-tsLA-1 strain (*pkcA*-ts) and the ΔbckALA-1 strain (Δ*bckA*) were incubated in YT medium supplemented with 0.6 M KCl for indicated times at 42°C. Arrowheads indicate the fluorescence of Lifeact-EGFP observed as filamentous structures in cytoplasm. Arrows indicate the fluorescence of Lifeact-EGFP observed as spots at the cortex of the tips of germ tube. Bar, 5 µm.

To examine the effect of PkcA inactivation on F-actin distribution during germination, we constructed a *pkcA*-ts mutant and a *bckA* deletion mutant in which Lifeact-EGFP was expressed under the control of *alcA*(p) (pkcA-tsLA-1 and ΔbckALA-1, respectively), and observed Lifeact-EGFP under the *alcA*(p)-inducing conditions supplemented with 0.6 M KCl at 42°C. In this case, we used KCl as an osmotic stabilizer instead of sorbitol to reduce background fluorescence. In the wild-type strain, Lifeact-EGFP was observed as spots at the cortex of the tip of the germ tube and as filamentous structures in the cytoplasm during germination (Fig. 8). In the *pkcA*-ts mutant, Lifeact-EGFP was observed as punctate structures in the cytoplasm after 6 h of incubation (Fig. 8A). However, this Lifeact-EGFP fluorescence was weaker than that of the wild-type strain (Fig. S5), and the cytoplasmic filamentous distribution of Lifeact-EGFP was not observed (Fig. 8). After 8 h of incubation, Lifeact-EGFP fluorescence dispersed in the cytoplasm and did not exhibit any polarized distribution (Fig. 8). These observations suggest that PkcA is required for polarity establishment during germination at 42°C. In the *bckA* deletion mutant, Lifeact-EGFP was observed as spots at the cortex of the tip of the germ tube and as filamentous structures in the cytoplasm (Fig. 8). This Lifeact-EGFP distribution in the *bckA* deletion mutant was similar to that of the wild-type strain. This observation suggests that the MAP kinase cascade containing BckA was not required for polarity establishment during germination at 42°C.

### PkcA is Required for Repolarization in Hyphae during Heat Stress

**Figure 9:**
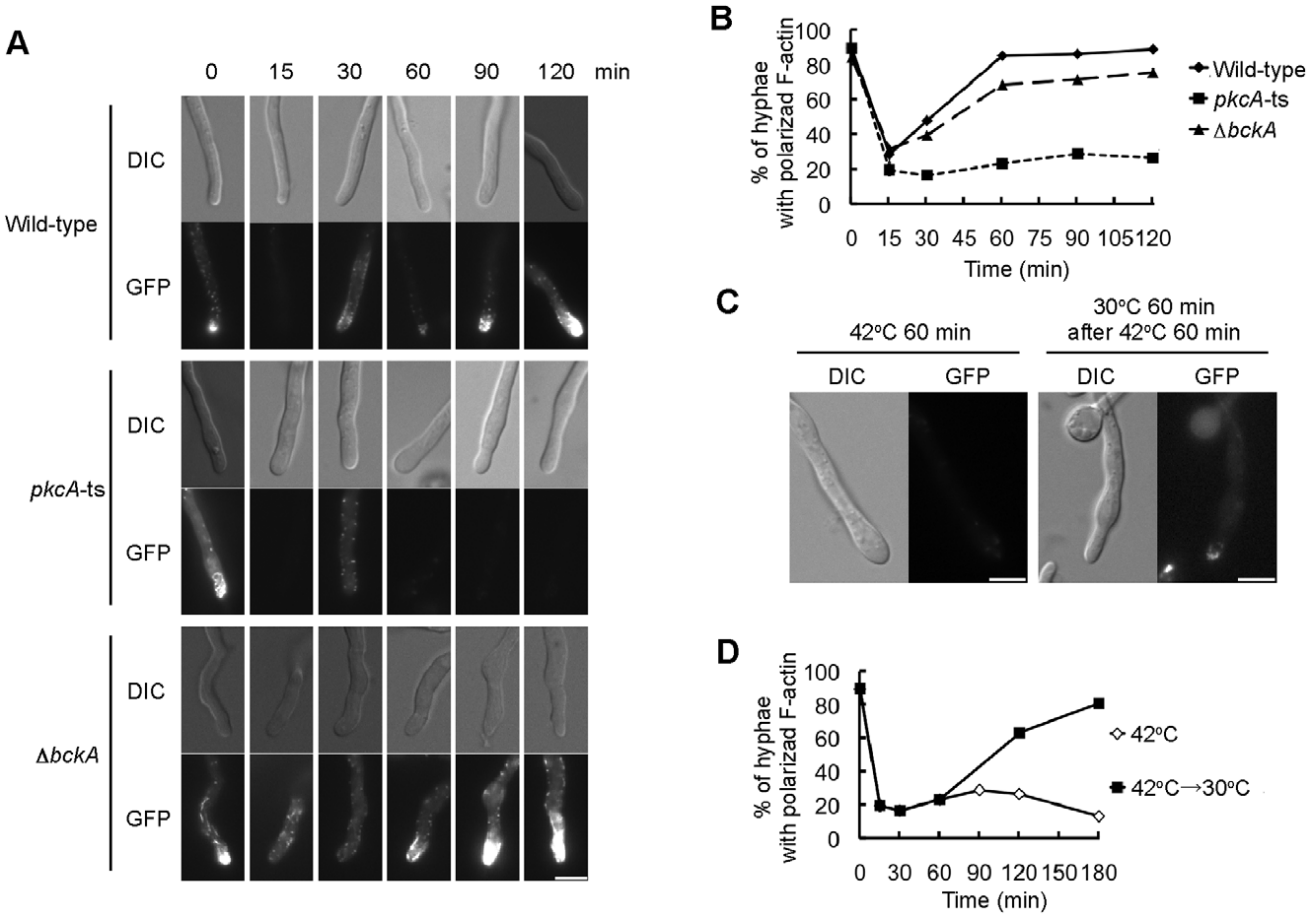
*pkcA* is required for the repolarization after the depolarization by heat stress. A. Conidia from the A1149LA-1 strain (Wild-type), the pkcA-tsLA-1 strain (*pkcA*-ts) and the ΔbckALA-1 strain (Δ*bckA*) were incubated in the YT medium on coverslips for 16 h (A1149LA-1 and pkcA-tsLA-1) or 24 h (ΔbckALA-1) at 30°C, shifted to 42°C, and observed after indicated time periods. Bar, 5 µm. B. The percentage of hyphae in which Lifeact-EGFP was observed at the tips was measured under the same condition as panel A. Data are shown as means ± S.E.M, of three independent experiments. C. Conidia from the pkcA-tsLA-1 strain were incubated in the YT medium on coverslips for 16 h at 30°C, shifted to 42°C, and observed 60 min after the shift (left panels). They were incubated for 60 min at 30°C after the incubation for 60 min at 42°C and observed (right panels). Bar, 5 µm. D. The percentage of hyphae in which Lifeact-EGFP was observed at the tips was measured when conidia of the pkcA-tsLA-1 strain incubated at 30°C were shifted to 42°C for indicated time periods (empty diamonds). The pkcA-tsLA-1 strain shifted to 30°C after the incubation for 60 min at 42°C was shown by filled rectangles.

To investigate whether PkcA was involved in polarity maintenance during hyphal growth, we observed the distribution of Lifeact-EGFP at the hyphal tips in the *pkcA*-ts mutant and the *bckA* deletion mutant grown at 30°C for 16 h and 20 h, respectively, and shifted to 42°C (Fig. 9A). After the shift to 42°C, the growth of the tips stopped, and the hyphal tips expanded in the *pkcA*-ts mutant. In the wild-type strain, spots and filamentous structures of Lifeact-EGFP disappeared within 15 min of the shift to 42°C, and then, they emerged at the hyphal tips 60 min after the shift (Fig. 9B). The fluorescence of Lifeact-EGFP, observed as spots, was clearly seen. Although the fluorescence observed as filamentous structures was very weak in that time, it was clearly observed at 120 min after the shift (Fig. 9A). In the *pkcA*-ts mutant, the fluorescence of Lifeact-EGFP disappeared within 15 min after the shift, similar to the wild-type strain, but subsequent emergence of the fluorescence of Lifeact-EGFP at the hyphal tips was not observed even 120 min after the shift (Fig. 9B). When the *pkcA*-ts mutant grown at 30°C was incubated at 42°C for 1 h and shifted to 30°C, the hyphal extension restarted at the expanded tips, and Lifeact-EGFP emerged to the tips (Fig. 9C and D). This result indicates that the *pkcA*-ts mutant was viable for at least 60 min after the shift to 42°C. In the *bckA* deletion mutant, the fluorescence of Lifeact-EGFP temporary disappeared at the tips and subsequently emerged at the hyphal tips after the shift to 42°C as observed in the wild-type strain (Fig. 9A and B). These results suggest that PkcA is required for repolarization after temporary depolarization at 42°C, while BckA is not required.

## Discussion

**Figure 10:**
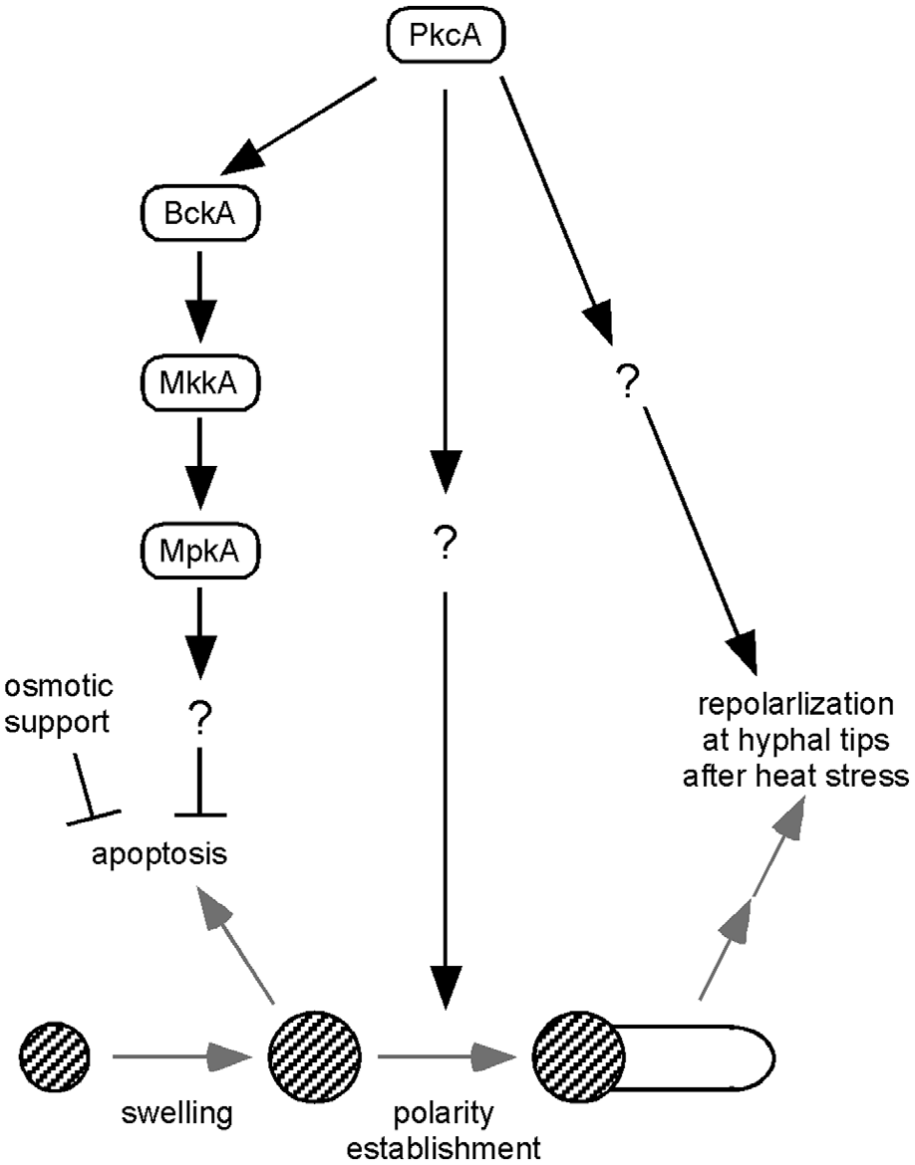
A model for the functions of PkcA at 42 °**C.** The functions of PkcA during germination and tip growth suggested in this study are shown in this figure. Hatched circles represent conidia. Involvement of MkkA in the signal transduction pathway for the suppression of apoptosis induction was not investigated in this study. “ ? ” means the possible presence of unknown factors.

Previous studies indicated that PKC is involved in several cellular processes and plays an important role in cell growth in yeasts and filamentous fungi. However, the functions of PKCs at the molecular level have not been well understood. In this study, we constructed *pkcA* temperature-sensitive mutants, and suggested that PkcA is involved in the suppression of apoptosis induction and in polarity establishment in *A. nidulans* under conditions of heat stress. A model for the functions of PkcA during germination and hyphal tip growth is shown in Figure 10.

In the previous study, we reported that *pkcA* is essential for the growth of *A. nidulans* [17]. The phosphorylation level of MpkA-FLAG dramatically decreased in the mutant at 42°C, indicating that PkcA was inactivated in this mutant at 42°C (Fig. 1A and D). When the *pkcA*-ts cells were incubated at 42°C, the DNA replication did not occur and the DNA degradation was induced. In addition, the *pkcA*-ts mutants, the *bckA* deletion mutant, and the *mpkA* deletion mutant exhibited apoptotic features such as ROS accumulation and DNA fragmentation at 42°C. These results suggest that the activation of MpkA is required for the suppression of apoptosis. When incubated with HU at 42°C, the wild-type strain did not exhibited apoptotic features (Fig. S2), suggesting that apoptosis was not induced by the defective DNA replication. Since these apoptotic features were largely suppressed by an osmotic stabilizer (Figs. 4A and 6A), it is possible that cell wall stress is involved in apotosis induction. The fact that treatment of micafungin slightly induced ROS accumulation in the mutant at 37°C (Fig. S3) may support this idea. In *S. cerevisiae*, the CWI pathway is activated under conditions of heat stress [30], [31]. During heat stress, trehalose accumulates in the cytoplasm resulting in increased turgor pressure, which is thought to activate the CWI pathway. Since orthologs of the genes involved in the pathway are all conserved in the *A. nidulans* genome, a similar activation mechanism is probably also present in *A. nidulans*. In *A. fumigatus*, it is suggested that farnesol, which induces apoptosis, inhibits the activation of the CWI pathway by delocalizing Rho1, which is an ortholog of Rho1p of *S. cerevisiae*, from the hyphal tip. In addition, deletion mutants of an *MKK1*/*2* ortholog or an *SLT2* ortholog in *A. fumigatus* are hypersensitive to farnesol [25], suggesting that the MAP kinase cascade is involved in farnesol-induced apoptosis in *A. fumigatus*. In this study, we showed that the pkcA-ts-2 mutant is hypersensitive to farnesol at 37°C and that the *alcA*(p)-*pkcA* mutant is hypersensitive to farnesol under *alcA*(p)-repressing condition. We also showed that the *bckA* deletion mutant and the *mpkA* deletion mutant are sensitive to farnesol (Fig. 5). These results suggest that PkcA and the MAP kinase cascade are required for the suppression of not only apoptosis induction under heat stress but also farnesol induced-apoptosis in *A. nidulans*. Colabardini *et al.* reported that a deletion mutant of *mpkA* was resistant to farnesol in *A. nidulans* [20]. The reason for this inconsistency is currently unclear. Since they incubated their *mpkA* deletion mutant on an YG plate containing 2% glucose, it is possible that the *mpkA* deletion mutant is resistant to farnesol at high glucose concentrations. Colabardini *et al.* also reported that an *alcA*(p)-*pkcA* strain was hypersensitive to farnesol under the *alcA*(p)-inducing condition [20]. Consistent with their result, our *alcA*(p)-*pkcA* strain was also hypersensitive to farnesol under the *alcA*(p)-inducing condition (Fig. 5B). Previously, we showed that the *alcA*(p)-*pkcA* strain was hypersensitive to several cell wall perturebing agents under *alcA*(p)-inducing condition [17]. These results suggest that proper expression level of *pkcA* is crucial for its function. In *A. fumigatus*, MpkA is involved in the induction of genes encoding a mycelial catalase (Cat1), a mitochondrial Mn superoxide dismutase (MnSOD), which contributes to protection against ROS generation, and a non-ribosomal peptide synthetase (NRPS), which contributes to tolerance against oxidative stress [32], [33]. This raises the possibility that the absence of activated MpkA leads to accelerated accumulation of ROS, resulting in apoptosis induction in *A. fumigatus* and *A. nidulans*.

Lifeact is shown to react to actin patches, cables, and rings without interfering with cellular functions in *N. crassa* [27], [28]. In this study, we showed that Lifeact also detects these actin structures without interfering with growth in *A. nidulans*. As shown in Fig. 8, the filamentous structure of Lifeact-EGFP was not observed in the *pkcA*-ts mutant incubated with the osmotic stabilizer at 42°C, suggesting that PkcA is required for the formation of filamentous structures of actin under heat stress. In *S. cerevisiae*, these structures of actin are nucleated by formins, Bni1p and Bnr1p [34]. High copy numbers of *BNI1* suppressed the growth defect of a *pkc1* temperature-sensitive mutant carrying the *stt1* allele [35]. In addition, it is suggested that Pkc1p is required for formin activation under conditions of heat stress [36]. In *A. nidulans*, the *sepA* gene encoding formin has been identified and is found to be essential for growth at high temperatures [37]. Thus, it is possible that PkcA is required for formin activation in *A. nidulans* and the *pkcA*-ts mutant does not grow due to its inability to activate SepA at high temperatures.

Since the fluorescence of the punctate structures of Lifeact-EGFP in the *pkcA*-ts mutant was weaker than that in the wild-type strain in the presence of the osmotic stabilizer at 42°C (Fig. 8), PkcA may be involved in the formation of punctate actin structures under conditions of heat stress. In *S. cerevisiae*, punctate actin structures are composed of highly branched actin filament networks and nucleated by the Arp2p/3p complex [34]. Since their orthologs are conserved in *A. nidulans*, it is possible that PkcA is also involved in the regulation of these orthologs.

In the previous study, we reported that the *pkcA* ^−^ conidia obtained from the *pkcA^+^*/*pkcA* ^−^ heterokaryon did not form colonies at 37°C. However, they germinated, and their germ tubes exhibited abnormal morphologies and frequently lysed [17]. Interestingly, the similarly obtained *pkcA* ^−^ conidia germinate and exhibit similar phenotypes in the presence of the osmotic stabilizer at 42°C (our unpublished data). The amount of PkcA remaining in the *pkcA* ^−^ conidia from the parental heterokaryon may be enough to establish the polarity during germination.

When the *pkcA*-ts mutant grown at 30°C was shifted to 42°C, actin structures at the hyphal tips disappeared and subsequent emergence of actin structures was not observed (Fig. 9). This suggests that PkcA is required for repolarization at the hyphal tips under conditions of heat stress. In *S. cerevisiae*, Pkc1p mediates depolarization during heat stress, which is not dependent on the MAP kinase cascade in the CWI pathway. In contrast, repolarization during heat stress depends on Mpk1p, which phosphorylates Rom2p, an upstream component of the CWI pathway and downregulates the pathway [38]. The results obtained in this study indicate that PkcA is required for repolarization in the hyphae, and that this process is independent of the MAP kinase cascade in the CWI pathway. We suggest that, during heat stress, the role of PKC in the repolarization of hyphae of *A. nidulans* differs from that observed in *S. cerevisiae* cells.

Previous studies indicate that PkcA plays a role in cell wall integrity, conidiation, and secondary metabolism. In this study, we suggest that PkcA is involved in suppression of apoptosis induction and polarity establishment under conditions of heat stress. Identification of the factors related to the functions of PkcA will help clarify the role of PkcA at the molecular level.

## Materials and Methods

### Strains and Growth Conditions

The *A. nidulans* strains used in this study are listed in Table 1. Minimal medium (MMG) was prepared as described by Rowlands and Turner [39]. In MMG, 2% glucose was added as a sole carbon source. In MMTF, 100 mM threonine and 0.1% fructose were added as carbon sources instead of glucose in MMG. YG medium (0.5% yeast extract, 1% glucose, 0.1% trace elements), YT medium (100 mM threonine was used instead of glucose in YG) and YTF medium (100 mM threonine and 0.1% fructose were used instead of glucose in YG) were also used. MMTF, YT and YTF were used to induce *alcA* expression. For the growth of *pyroA4*, and *riboB2* mutants, 0.5 µg/ml pyridoxine and 2.5 µg/ml riboflavin, respectively, were added to MMG or MMTF. For the growth of *pyrG89* mutants, 10 mM uridine and 10 mM uracil were added to MMG, MMTF, YG, YT or YTF. Solid media were prepared by adding 1.5% agar. Hydroxyurea (Nacalai tesque), calcofluor white (Sigma), congo red (Wako), and micafungin (a gift of Astellas Pharma) were used. Bacterial and fungal transformations were performed as described previously [40].

### Plasmid Constructs and Oligonucleotides

Oligonucleotides used in this study are provided as *Supporting information* (Table S1). To create the *bckA* (*AN4887*) deletion mutants, we constructed plasmids pBSbckA, pBSbckAΔ, and pbckA::pyroA as follows: A primer set of 5bckAF and 3bckAR-2 was used to amplify a 9.9-kb fragment containing *bckA* from the total DNA of the A26 strain. We confirmed that no mutation was introduced into the sequence in the cloned PCR product. The fragment was digested with *Hin*dIII and *Sca*I, and ligated with *Hin*dIII- and *Sma*I-digested pBluescript II SK^+^ (Stratagene) to yield pBSbckA. pBSbckAΔ that contains the upstream and downstream regions of *bckA* ORF was constructed by selfligating a 6.7-kb *Bsp*1407I fragment of pBSbckA. pbckA::pyroA was constructed by inserting a 2.3-kb *Aor*51HI-*Bst*1107I fragment of pUCpyroA2 (Tanaka A., Ohta A., Horiuchi H., unpublished) containing *pyroA* into the *Bst*1107I site of pBSbckAΔ.

We constructed plasmids pBSpyrG as follows: A primer set of pyrGF and pyrGR was used to amplify a 4.5-kb fragment containing *pyrG* from the total DNA of A26 strain, and the fragment was digested with *Bgl*II and *Eco*RV and ligated with *Bgl*II- and *Eco*RV-digested pBluescrpt II SK^+^ to yield pBSpyrG. To create strains in which Lifeact-EGFP was expressed under the control of the *alcA*(p), we used the fusion PCR method [41]. A 2.5-kb fragment containing the *alcA*(p) was amplified from total DNA of the alcA(p)-pkcA-3 strain using primers, riboBF and alcApR. A coding region of N-terminal 17 aa of Abp140p was amplified from the total DNA of *S. cerevisiae* W303 strain (*MAT*a *ade2-1 ura3-1 his3–11*,*15 trp1-1 leu2–3*,*112 can1–100*) using primers alcA(p)-abp140F and abp140R-link2. A coding region of EGFP was amplified from pEGFP (Clontech Laboratories Inc.) using primers, link2-egfpF and BamHI-egfpR. These amplified fragments were fused by the fusion PCR method using primers riboBF and BamHI-egfpR. ppyrGLA was constructed by ligating the 1.5-kb *Bam*HI-*Nde*I fragment of the fused fragment into the *Bam*HI-*Nde*I site of pBSpyrG. To create the strains in which MpkA tagged with three repeats of FLAG epitopes at its C-terminus (MpkA-FLAG) was expressed from its own promoter, we constructed plasmids pMAPKsh and pMPKA-pyroA as follows: An 8.7-kb fragment containing *mpkA* that was amplified from the total DNA of A26 strain using primers, mpkA.s and mpkA.as, was digested with *Pst*I, and was ligated with *Pst*I-digested pUC18 to yield pMAPKsh. pMPKA-pyroA containing *mpkA* and *pyroA* was constructed by inserting 1.9-kb *Afl*II fragment from pUCpyroA2 into the *Afl*II site of pMPKAsh.

### Construction of *A. nidulans* Strains by Transformation

Detailed construction procedures are described in *Supporting information* (Text S1 and Fig. S6).

### Construction of the pkcA-ts nkuA^+^-1 Strain and the pkcA-ts nkuA^+^-2 Strain by Genetic Cross

We constructed the pkcA-ts nkuA^+^-1 and pkcA-ts nkuA^+^-2 strains as follows: Conidia of the ABPU1 strain and the pkcA-ts-2 mutant were mixed, inoculated on the YG plate, and incubated for 3 days at 30°C. The plate was seeled and incubated for 2 weeks 30°C. Ascospores from the cleistothecium formed on the YG plate were plated on the selective medium and incubated for 3 days at 30°C. Strains, which were auxotrophic for arginine and did not grow at 42°C, were selected and designated pkcA-ts nkuA^+^-1 and pkcA-ts nkuA^+^-2.

### Determination of Conidiation Efficiency

Conidiation efficiency was determined as follows: 10^3^ conidia were spotted on the YG plate and incubated for 72 h. All conidia were harvested from the colony and counted the number. The number of conidia divided by the colony diameter was defined as the conidiation efficiency.

### Determination of Viability

Conidia were incubated in the YG medium for the indicated times at 42°C, and aliquots containing 10^2^ of the conidia were plated on the YG medium and incubated for 24 h at 30°C. The number of colonies formed was counted and defined as the viability.

### Preparation of Cell Lysate and Western Blot Analysis

Total cell extracts were prepared by homogenizing mycelia after freezing with liquid nitrogen, and they were suspended in 200 µl extraction buffer (0.8 M sucrose, 10 mM Tris-HCl, pH 8.2), incubated for 5 min at 100°C and followed by centrifugation at 5000 g for 5 min at 4°C to remove cellular debris. Samples were separated by electrophoresis on 10% polyacrylamide gel containing sodium dodecyl sulfate [42] and electroblotted by Biotrace™ Nitrocellulose transfer membrane (Pall Corporation). To detect MpkA-FLAG, the membranes were incubated with a mouse anti-FLAG antibody (Sigma) as a primary antibody at a 1∶2000 dilution and with a horseradish peroxidase (HRP)-conjugated anti-mouse IgG antibody (Cell Signaling Technology) as a secondary antibody at a 1∶5000 dilution. To detect phosphorylated MpkA-FLAG, the membranes were incubated with a rabbit anti-phospho-p44/42 MAPK antibody (Cell Signaling Technology) as a primary antibody at a 1∶1000 dilution and with HRP-conjugated anti-rabbit IgG antibody (Cell Signaling Technology) as a secondary antibody at a 1∶5000 dilution. The HRP was visualized with ECL Western Blotting Detection System (GE healthcare) as recommended by manufacture’s instructions. Can Get Signal immunoreaction enhancer solution (Toyobo) was used when necessary.

### Fluorescence Microscopy

Staining of nuclei was carried out as follows: Cells were fixed overnight with 70% ethanol, washed twice with phosphate-buffered saline (PBS; 13.7 mM NaCl, 2.7 mM KCl, 8.1 mM NaH_2_PO_4_, 1.5 mM KH_2_PO_4_), and stained for 5 min at room temperature with a solution containing 50 µg/ml of DAPI (Wako). Conidia and mycelia were observed under an Olympus BX52 fluorescence microscope. Images were taken and analyzed with ORCA-ER charge-coupled device camera (Hamamatsu) and Aquacosmos software (Hamamatsu).

### Flow Cytometric Analysis

Conidia were incubated in the YG medium at 42°C, fixed overnight with 70% ethanol, and washed with 50 mM sodium citrate. They were incubated for 4 h at 37°C in 500 µl of 50 mM sodium citrate containing 10 µg/ml RNase A. Then, 500 µl of 50 mM sodium citrate containing 16 µg/ml propidium iodide was added and incubated at least for 1 h at 4°C. The samples were subjected to a FACStar fluoreascence-activated cell sorter (Becton Dickinson & Co. Mountaion View).

### Reactive Oxygen Species Detection

Intracellular ROS levels were monitored with the oxydant-sensitive probe 2′,7′-dichlorodihydrofluorescein diacetate (H_2_DCFDA, Molecular Probes). Conidia were incubated in the YG medium for 4 h 42°C. They were washed with distilled water, suspended in a solution containing 0.01 mM H_2_DCFDA, and then incubated for 3 h at 30°C. They were washed twice with distilled water, suspended in 20 µl MOPS7 buffer (0.1 M MOPS, pH 7.0), and observed by fluorescence microscopy.

### TUNEL Assay

DNA strand breaks were detected using In Situ Cell Death Detection Kit, fluorescein (Roche Diagnostics). Conidia were incubated in the YG medium on coverslips for 8 h at 42°C. The coverslips were washed three times with PBS, and then overlaid for 1 h at room temperature with 2% paraformaldehyde solution. The coverslips were washed twice with PBS and then overlaid for 1 h at room temperature with 200 µl PBS containing 0.6 mg Yatalase (TaKaRa Bio Inc.), 0.2 mg lysing enzyme L2265 (Sigma) and 2 mg egg white (Sigma). The coverslips were washed twice with PBS, overlaid for 2 min on ice with permeabilisation solution (0.1% TritonX-100, 0.1% sodium citrate), washed twice with PBS, and then overlaid for 1 h at 37°C in dark with TUNEL reaction mixture (Roche Diagnostics). The coverslips were washed twice with PBS, overlaid for 5 min at room temperature with a solution containing 50 µg/ml of DAPI, washed twice with PBS, and observed by fluorescence microscopy.

### Cytochalasin A Treatment

Conidia were incubated on coverslips in 100 µl of the MMTF medium for 16 h at 37°C, 5 µl of 200 µg/ml Cytochalasin A (MP Biomedicals) was added to the medium, and the coverslips were incubated for 15 min at 37°C and observed by fluorescence microscopy.

## Supporting Information

Figure S1 **The phosphorylation level of MpkA-FLAG in the** ***pkcA*** **-ts mutant under the condition with the osmotic stabilizer.** Cell extracts were prepared from A1149/MF-1 (Wild-type) and pkcA-ts/MF-1 (*pkcA*-ts), which were incubated on the MMG plate supplemented with 1.2 M sorbitol for 20 h at 30°C and incubated for indicated time periods at 42°C. The upper panel shows the results of immunoblotting with anti-phospho-p44/42 MAPK antibody (P-MpkA-FLAG). The lower panel shows the results of immunoblotting with anti-FLAG antibody (MpkA-FLAG). The numbers under the lower panel indicate the ratio of MpkA-FLAG phosphorylation at the indicated time periods comparing to that in the wild-type strain at 0 h.
(TIF)

Figure S2 **The effect of HU treatment on the** ***pkcA*** **-ts mutant at 42°C.** A. Conidia from the A1149 strain (Wild-type) and the pkcA-ts-2 mutant (*pkcA*-ts) were incubated in the YG medium containing 100 mM HU for indicated times at 42°C, and analysed by flow cytometry. B. Conidia from the A1149 strain (Wild-type) and the pkcA-ts-2 mutant (*pkcA*-ts) were incubated in the YG medium containing 100 mM HU for 4 h at 42°C, and treated with H_2_DCFDA. Bar, 10 µm. C. The ratios of cells stained with H_2_DCFDA under the conditions of panel B. Data are shown as means ± S.E.M. of three independent experiments. D. Conidia from the A1149 strain (Wild-type) and the pkcA-ts-2 mutant (*pkcA*-ts) were inoculated in the liquid YG medium containing 100 mM HU on the coverslips, incubated for 8 h at 42°C and fixed, labeled with TUNEL and stained with DAPI. Bar, 10 µm.
(TIF)

Figure S3 **The effects of cell wall stresses and ER stress on the** ***pkcA*** **-ts mutant at 37°C.** A. Conidia from the A1149 strain (Wild-type) and the pkcA-ts-2 mutant (*pkcA*-ts) were incubated in the YG medium for indicated times at 37°C, and analyzed by flow cytometry. B. Conidia from the A1149 strain (Wild-type) and the pkcA-ts-2 mutant (*pkcA*-ts) were incubated in the YG medium for 4 h at 42°C, and treated with H_2_DCFDA. Conidia from the A1149 strain pretreated with farnesol were treated with H_2_DCFDA and indicated as control. Bar, 10 µm. C. The ratios of cells stained with H_2_DCFDA under the conditions of panel B. Data are shown as means ± S.E.M. of three independent experiments. D. Conidia from the A1149 strain (Wild-type) and the pkcA-ts-2 mutant (*pkcA*-ts) were inoculated in the liquid YG medium on the coverslips, incubated for 8 h at 37°C and fixed, labeled with TUNEL and stained with DAPI. Bar, 10 µm. E. Conidia from the A1149 strain (Wild-type) and the pkcA-ts-2 mutant (*pkcA*-ts) were incubated in the YG medium containing 100 µg/ml calcofluor white (CFW), 100 µg/ml congo red (CR), 1 µg/ml micafungin, or 10 mM dithiothreitol (DTT) for 4 h at 42°C, and treated with H_2_DCFDA. F. The ratios of cells stained with H_2_DCFDA under the conditions of panel E. Data are shown as means ± S.E.M. of three independent experiments.
(TIF)

Figure S4 **The number of nuclei in the** ***pkcA*** **-ts mutant under heat stress condition.** Conidia from the A1149 strain (Wild-type) and the pkcA-ts-2 mutant (*pkcA*-ts) were incubated in the YG medium supplemented with 1.2 M sorbitol for 9 h at 42°C, fixed, stained with DAPI, and the number of nuclei was measured (n = 150). Data are shown as means ± S.E.M. of three independent experiments.
(TIF)

Figure S5 **Lifeact-EGFP fluorescence of the** ***pkcA*** **-ts mutant and the Δ** ***bckA*** **deletion mutant taken under the same image acquisition condition.** Conidia from the A1149LA-1 strain (Wild-type), the pkcA-tsLA-1 strain (*pkcA*-ts) and the ΔbckALA-1 strain (Δ*bckA*) were incubated in the YT medium supplemented with 0.6 M KCl for 6 h at 42°C. The pictures of EGFP were taken under the same image acquisition condition. Bar, 10 µm.
(TIF)

Figure S6 **Constructions of the** ***pkcA*** **-ts mutants, the** ***alcA*** **(p)-** ***pkcA*** **mutants, the** ***bckA*** **deletion mutants, the** ***mpkA*** **deletion mutants, and the strains in which Lifeact-EGFP was expressed under the control of** ***alcA*** **(p).** A. A scheme for the replacement of Pro959 with Leu in PkcA. A 0.9-kb fragment amplified from the total DNA of the A26 strain using primers, 3pkcA518F-friboB and 3pkcAR1405R-n, was used as a probe. Asterisks indicate the mutation point of the *pkcA*-ts allele. B. Southern analysis of genomic DNAs of the A1145 strain (wild-type, lane 1) and the pkcA-ts-2, −3, and −5 mutants (the *pkcA*-ts mutants, lanes 2, 3, and 4, respectively) digested with *Bgl*II. The probe indicated in panel A detected 2.0-kb band in lane 1, while the same probe detected 4.1-kb bands in lanes 2, 3, and 4. C. A scheme for insertion of the *alcA* promoter into the gonome locus in front of the *pkcA* initiation codon. A 1.0-kb fragment amplified from the total DNA of the A26 strain using primers, 5npkcAF and 5pkcAR-friboB, was used as a probe. D. Southern analysis of genomic DNAs of the A1145 strain (wild-type, lane 1) and the alcA(p)-pkcA-3 and −4 mutants (the *alcA*(p)-*pkcA* mutants, lanes 2 and 3, respectively) digested with *Pst*I and *Xho*I. The probe indicated in C detected 1.8-kb band in lane 1, while the same probe detected 3.7-kb bands in lanes 2 and 3. E. A scheme for the deletion of the *bckA* gene. A 0.8-kb *Bst*1107I-*Hin*dIII fragment from pBSbckAΔ was used as a probe. F. Southern analysis of genomic DNAs of the A1149 strain (wild-type, lane 1) and the ΔbckA-1 and-2 mutants (the *bckA* deletion mutants, lanes 2 and 3, respectively) digested with *Pst*I. The probe indicated in E detected 2.0-kb band in lane 1, while the same probe detected 3.7-kb bands in lanes 2 and 3. G. A scheme for the deletion of the *mpkA* gene. A 1.1-kb fragment amplified from the A26 strain using primers, 3mpkAF-fpyrG and 3mpkAR-n, was used as a probe. H. Southern analysis of genomic DNAs of the A1149 strain (wild-type, lane 1) and the ΔmpkA-1, -2, and -8 mutants (the *mpkA* deletion mutants, lanes 2, 3, and 4, respectively) digested with *Stu*I. The probe indicated in G detected 1.9-kb band in lane 1, while the same probe detected 2.9-kb bands in lanes 2, 3, and 4. I. A scheme for insertion of *alcA* promoter and *lifeact-egfp* into the genome locus in the rear of the *pyrG* gene. A 0.8-kb fragment amplified from the total DNA of the A26 strain using primers, pyrG5 and pyrG3, was used as a probe. J. Southern analysis of genomic DNAs of the A1149 strain (wild-type, lane 1) and the A1149LA-1, 2 mutants (the A1149LA mutants, lanes 2 and 3, respectively) digested with *Eco*RV. The probe indicated in I detected 4.7-kb band in lane 1, while the same probe detected 6.3-kb bands in lanes 2 and 3.
(TIF)

Table S1 **Oligonucleotides used in this study.**
(DOC)

Text S1 **Detailed description of** ***A. nidulans*** **strain constructions.**
(DOC)
