# Supplementary figures and images for: Involvement of Protein Kinase C in the Suppression of Apoptosis and in Polarity Establishment in Aspergillus nidulans under Conditions of Heat Stress
Source: PLoS One. 2012 Nov 28;7(11):e50503. doi: 10.1371/journal.pone.0050503 (PMC3509046; doi:10.1371/journal.pone.0050503)

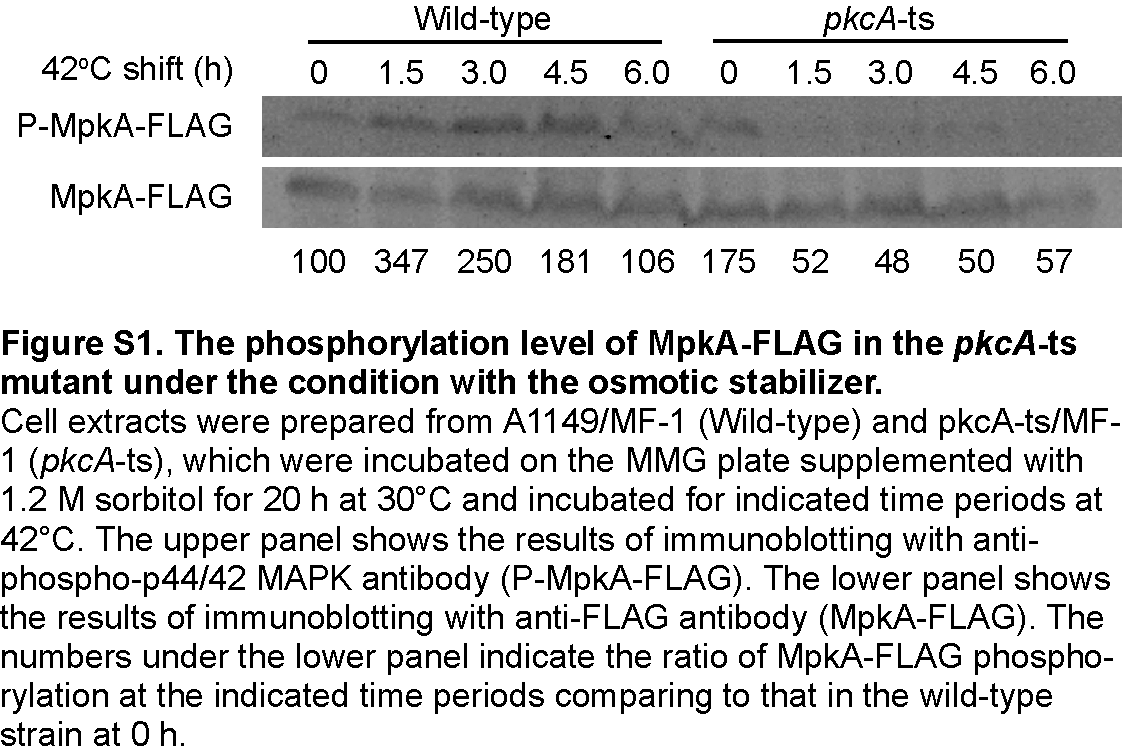

Supplement: Figure S1 — The phosphorylation level of MpkA-FLAG in the pkcA -ts mutant under the condition with the osmotic stabilizer. Cell extracts were prepared from A1149/MF-1 (Wild-type) and pkcA-ts/MF-1 (pkcA-ts), which were incubated on the MMG plate supplemented with 1.2 M sorbitol for 20 h at 30°C and incubated for indicated time periods at 42°C. The upper panel shows the results of immunoblotting with anti-phospho-p44/42 MAPK antibody (P-MpkA-FLAG). The lower panel shows the results of immunoblotting with anti-FLAG antibody (MpkA-FLAG). The numbers under the lower panel indicate the ratio of MpkA-FLAG phosphorylation at the indicated time periods comparing to that in the wild-type strain at 0 h. (TIF) [file pone.0050503.s001.tif]

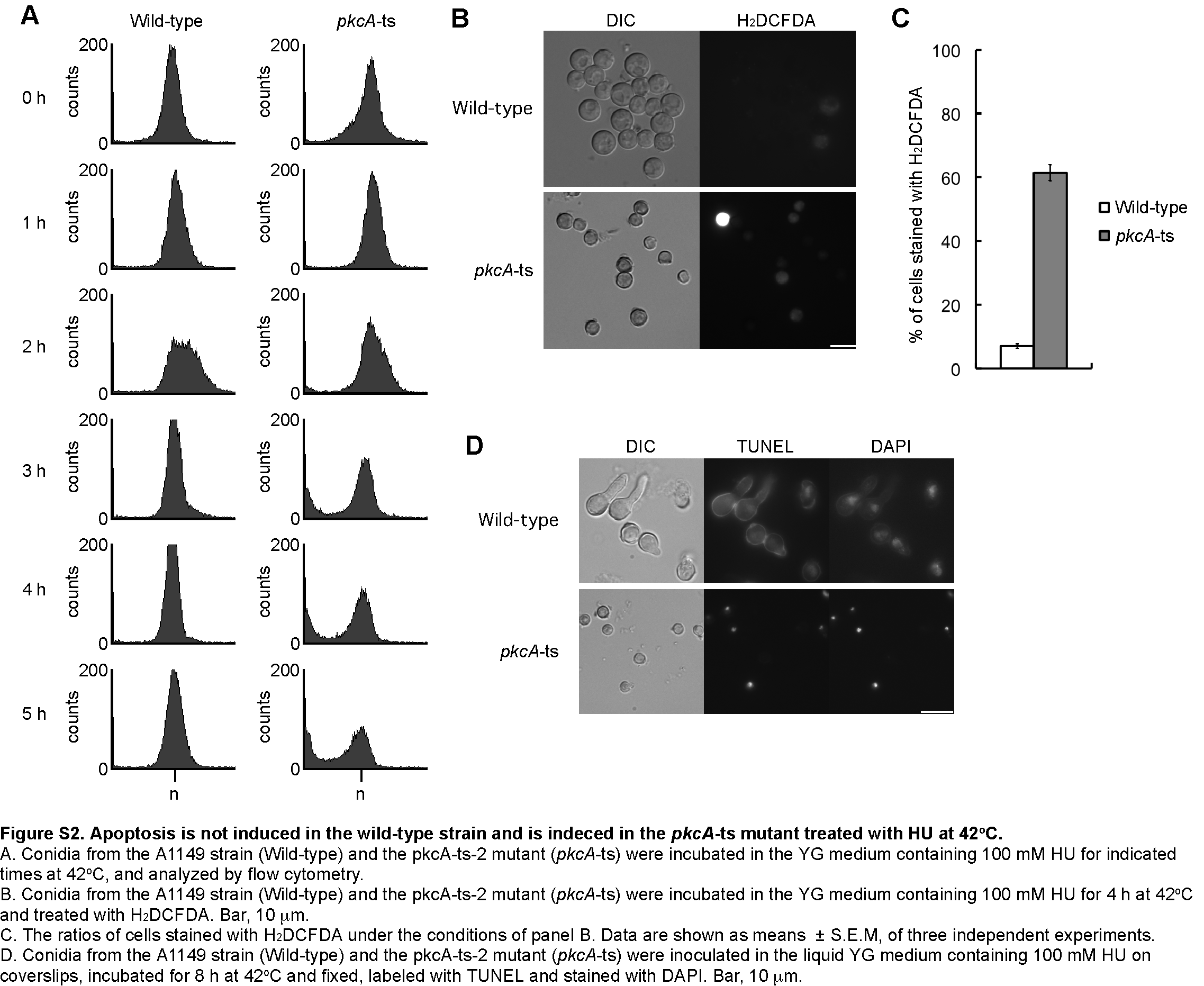

Supplement: Figure S2 — The effect of HU treatment on the pkcA -ts mutant at 42°C. A. Conidia from the A1149 strain (Wild-type) and the pkcA-ts-2 mutant (pkcA-ts) were incubated in the YG medium containing 100 mM HU for indicated times at 42°C, and analysed by flow cytometry. B. Conidia from the A1149 strain (Wild-type) and the pkcA-ts-2 mutant (pkcA-ts) were incubated in the YG medium containing 100 mM HU for 4 h at 42°C, and treated with H2DCFDA. Bar, 10 µm. C. The ratios of cells stained with H2DCFDA under the conditions of panel B. Data are shown as means ± S.E.M. of three independent experiments. D. Conidia from the A1149 strain (Wild-type) and the pkcA-ts-2 mutant (pkcA-ts) were inoculated in the liquid YG medium containing 100 mM HU on the coverslips, incubated for 8 h at 42°C and fixed, labeled with TUNEL and stained with DAPI. Bar, 10 µm. (TIF) [file pone.0050503.s002.tif]

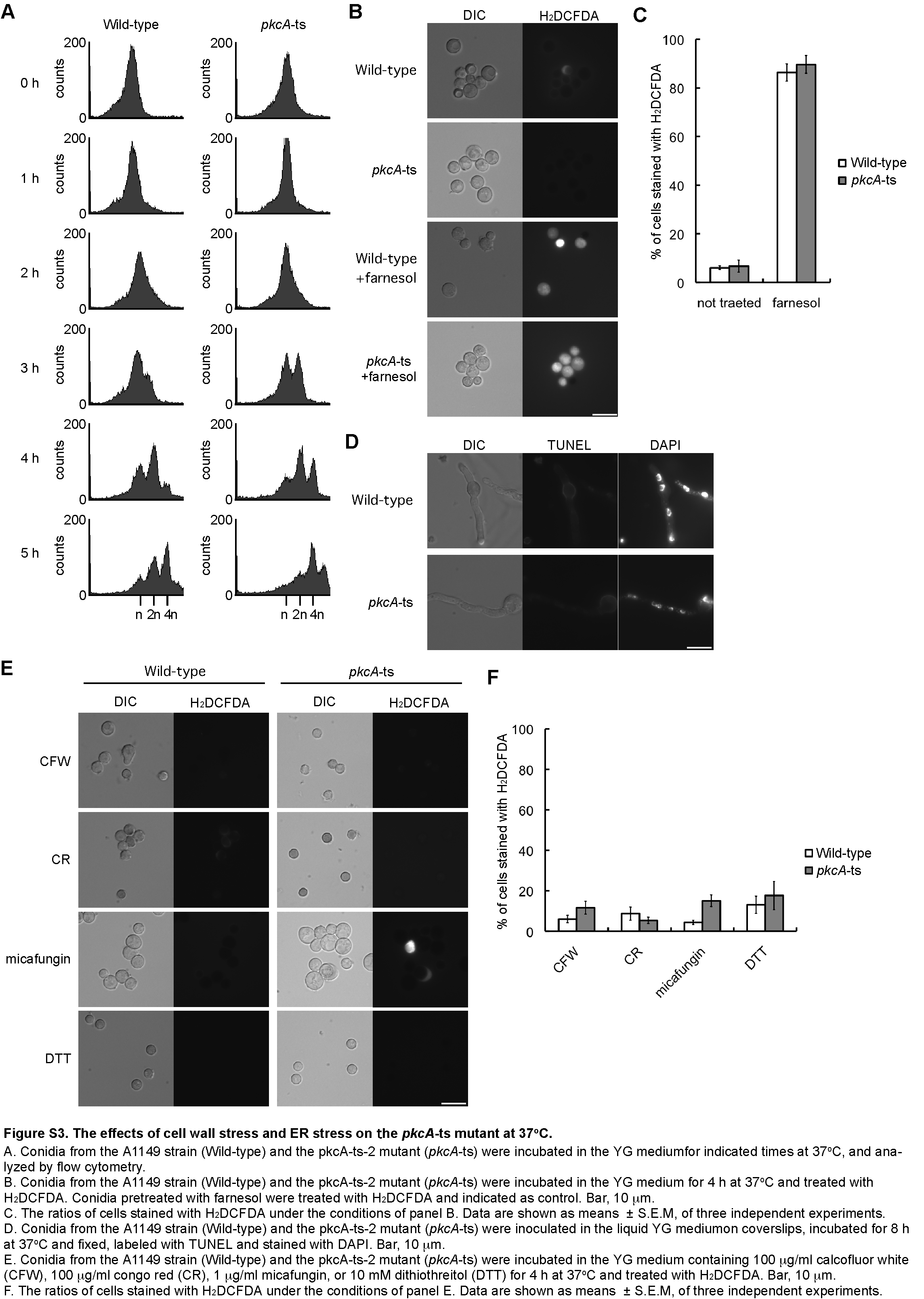

Supplement: Figure S3 — The effects of cell wall stresses and ER stress on the pkcA -ts mutant at 37°C. A. Conidia from the A1149 strain (Wild-type) and the pkcA-ts-2 mutant (pkcA-ts) were incubated in the YG medium for indicated times at 37°C, and analyzed by flow cytometry. B. Conidia from the A1149 strain (Wild-type) and the pkcA-ts-2 mutant (pkcA-ts) were incubated in the YG medium for 4 h at 42°C, and treated with H2DCFDA. Conidia from the A1149 strain pretreated with farnesol were treated with H2DCFDA and indicated as control. Bar, 10 µm. C. The ratios of cells stained with H2DCFDA under the conditions of panel B. Data are shown as means ± S.E.M. of three independent experiments. D. Conidia from the A1149 strain (Wild-type) and the pkcA-ts-2 mutant (pkcA-ts) were inoculated in the liquid YG medium on the coverslips, incubated for 8 h at 37°C and fixed, labeled with TUNEL and stained with DAPI. Bar, 10 µm. E. Conidia from the A1149 strain (Wild-type) and the pkcA-ts-2 mutant (pkcA-ts) were incubated in the YG medium containing 100 µg/ml calcofluor white (CFW), 100 µg/ml congo red (CR), 1 µg/ml micafungin, or 10 mM dithiothreitol (DTT) for 4 h at 42°C, and treated with H2DCFDA. F. The ratios of cells stained with H2DCFDA under the conditions of panel E. Data are shown as means ± S.E.M. of three independent experiments. (TIF) [file pone.0050503.s003.tif]

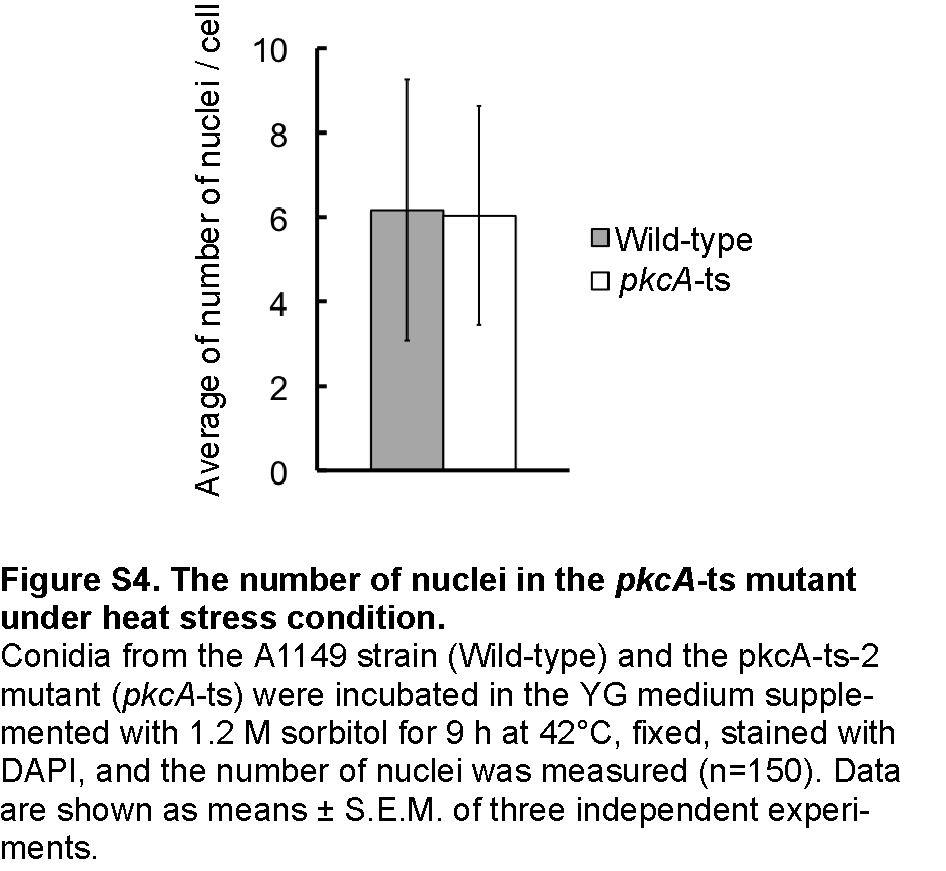

Supplement: Figure S4 — The number of nuclei in the pkcA -ts mutant under heat stress condition. Conidia from the A1149 strain (Wild-type) and the pkcA-ts-2 mutant (pkcA-ts) were incubated in the YG medium supplemented with 1.2 M sorbitol for 9 h at 42°C, fixed, stained with DAPI, and the number of nuclei was measured (n = 150). Data are shown as means ± S.E.M. of three independent experiments. (TIF) [file pone.0050503.s004.tif]

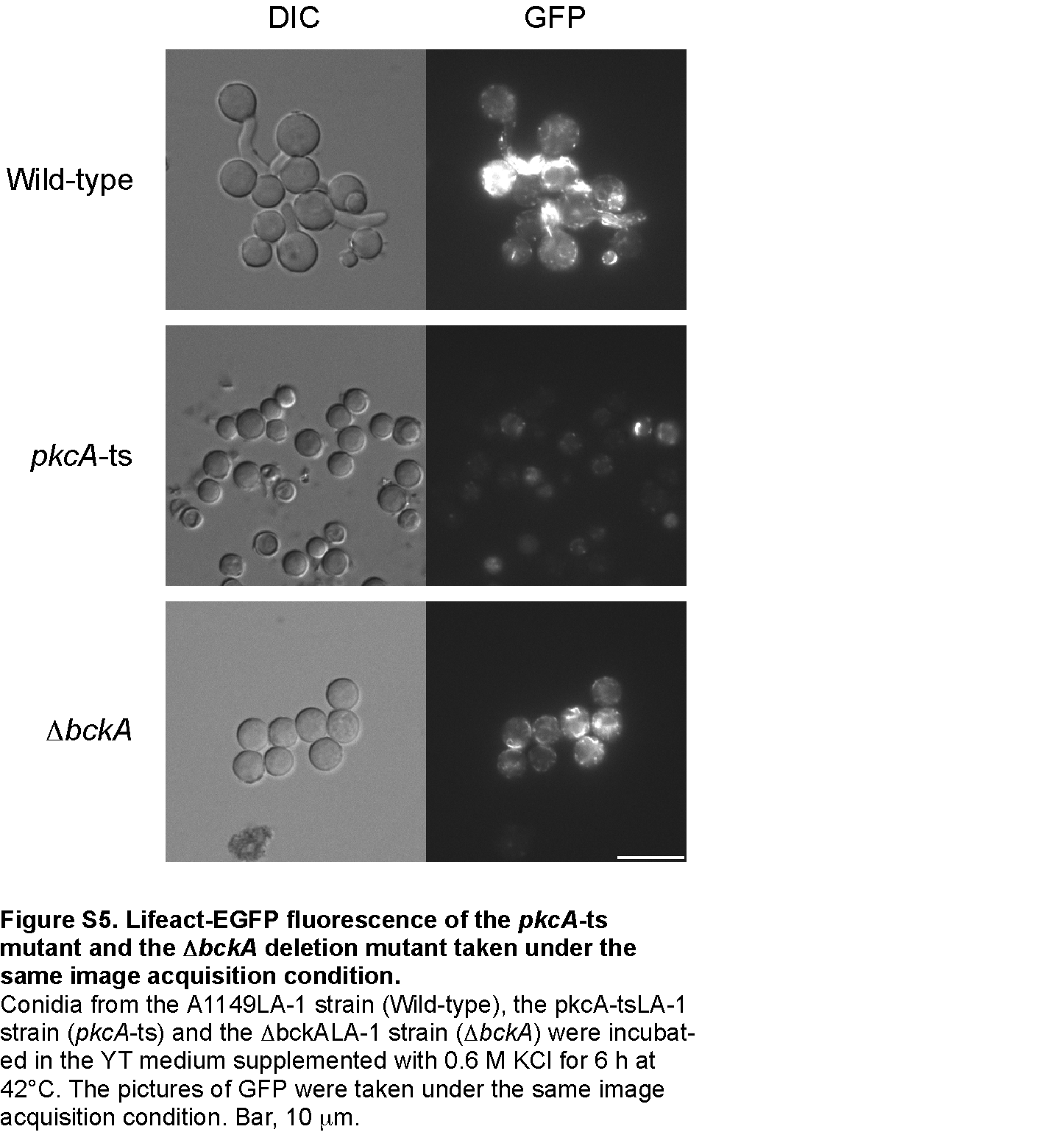

Supplement: Figure S5 — Lifeact-EGFP fluorescence of the pkcA -ts mutant and the Δ bckA deletion mutant taken under the same image acquisition condition. Conidia from the A1149LA-1 strain (Wild-type), the pkcA-tsLA-1 strain (pkcA-ts) and the ΔbckALA-1 strain (ΔbckA) were incubated in the YT medium supplemented with 0.6 M KCl for 6 h at 42°C. The pictures of EGFP were taken under the same image acquisition condition. Bar, 10 µm. (TIF) [file pone.0050503.s005.tif]

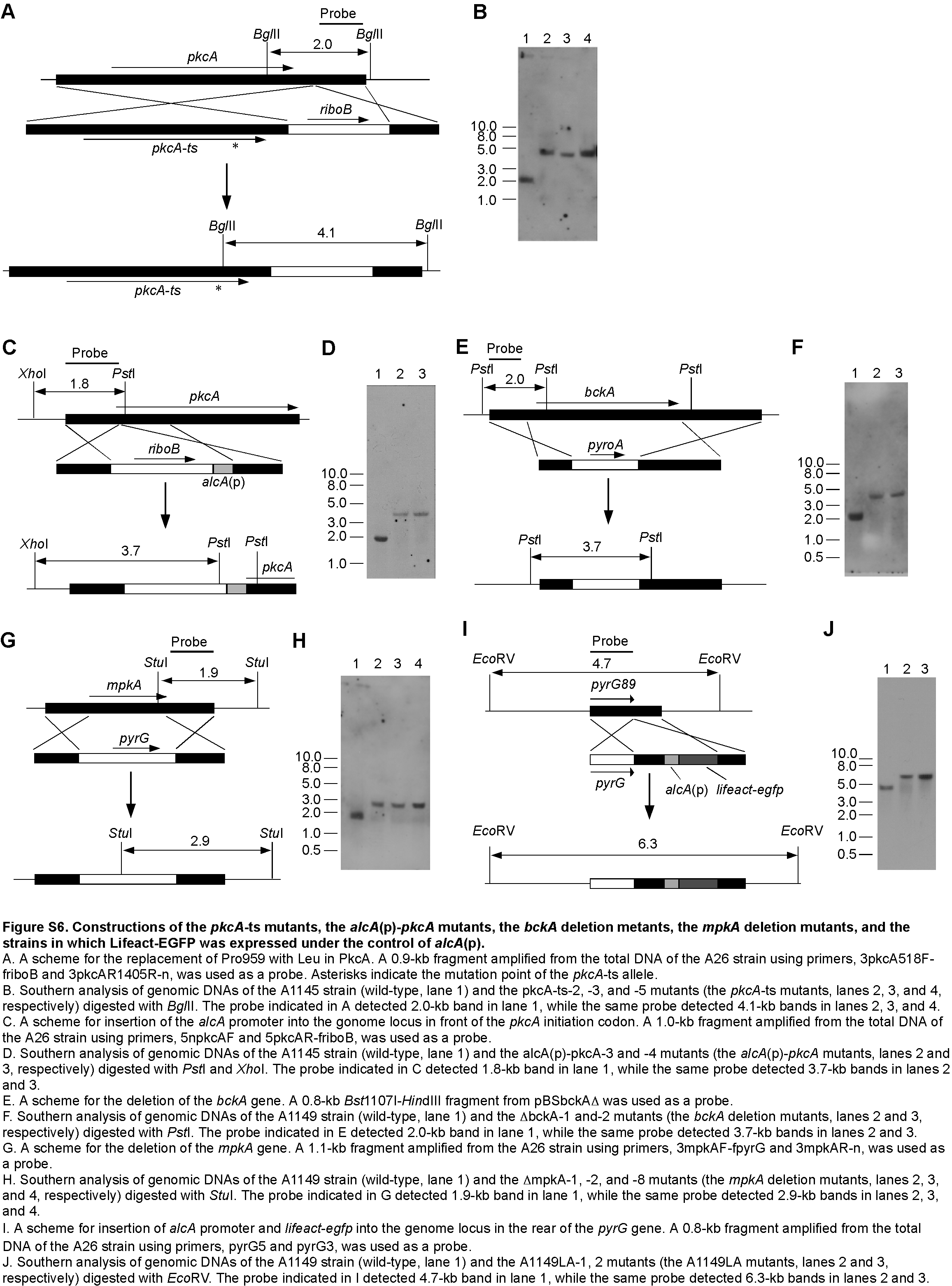

Supplement: Figure S6 — Constructions of the pkcA -ts mutants, the alcA (p)- pkcA mutants, the bckA deletion mutants, the mpkA deletion mutants, and the strains in which Lifeact-EGFP was expressed under the control of alcA (p). A. A scheme for the replacement of Pro959 with Leu in PkcA. A 0.9-kb fragment amplified from the total DNA of the A26 strain using primers, 3pkcA518F-friboB and 3pkcAR1405R-n, was used as a probe. Asterisks indicate the mutation point of the pkcA-ts allele. B. Southern analysis of genomic DNAs of the A1145 strain (wild-type, lane 1) and the pkcA-ts-2, −3, and −5 mutants (the pkcA-ts mutants, lanes 2, 3, and 4, respectively) digested with BglII. The probe indicated in panel A detected 2.0-kb band in lane 1, while the same probe detected 4.1-kb bands in lanes 2, 3, and 4. C. A scheme for insertion of the alcA promoter into the gonome locus in front of the pkcA initiation codon. A 1.0-kb fragment amplified from the total DNA of the A26 strain using primers, 5npkcAF and 5pkcAR-friboB, was used as a probe. D. Southern analysis of genomic DNAs of the A1145 strain (wild-type, lane 1) and the alcA(p)-pkcA-3 and −4 mutants (the alcA(p)-pkcA mutants, lanes 2 and 3, respectively) digested with PstI and XhoI. The probe indicated in C detected 1.8-kb band in lane 1, while the same probe detected 3.7-kb bands in lanes 2 and 3. E. A scheme for the deletion of the bckA gene. A 0.8-kb Bst1107I-HindIII fragment from pBSbckAΔ was used as a probe. F. Southern analysis of genomic DNAs of the A1149 strain (wild-type, lane 1) and the ΔbckA-1 and-2 mutants (the bckA deletion mutants, lanes 2 and 3, respectively) digested with PstI. The probe indicated in E detected 2.0-kb band in lane 1, while the same probe detected 3.7-kb bands in lanes 2 and 3. G. A scheme for the deletion of the mpkA gene. A 1.1-kb fragment amplified from the A26 strain using primers, 3mpkAF-fpyrG and 3mpkAR-n, was used as a probe. H. Southern analysis of genomic DNAs of the A1149 strain (wild-type, lane 1) and the Δmp [file pone.0050503.s006.tif]
