# Supplementary material for: Involvement of Protein Kinase C in the Suppression of Apoptosis and in Polarity Establishment in Aspergillus nidulans under Conditions of Heat Stress
Source: PLoS One. 2012 Nov 28;7(11):e50503. doi: 10.1371/journal.pone.0050503 (PMC3509046; doi:10.1371/journal.pone.0050503)
Supplement: Table S1 — Oligonucleotides used in this study. (DOC) [file pone.0050503.s007.doc]

**Table S1. Oligonucleotides used in this study.**

| **Primer name** | **sequence 5' to 3'** |
| --- | --- |
| 5bckAF | GAAGAGAGCTGACATGGAGAAAG |
| 3bckAR2 | TCTGAGTAGCCTTTGCCCC |
| pyrGF | CGAGATCTCCATCCCCG |
| pyrGR | TTCTGCAGGGTTGAGGAAG |
| riboBF | CTGTTGGAAGATAGATGAC |
| alcApR | GGTACCGCTAATTAACTG |
| alcA(p)-abp140 | CTACTCAGTTAATTAGCGGTACCATGGGTGTCGCAGATTT |
| abp140R-link2 | ACCACTACCAGTAGCACTCGCTTCTTCCTTTGAGATGCTTT |
| link2-egfpF | GCGAGTGCTACTGGTAGTGGTATGGTGAGCAAGGGCGA |
| BamHI-egfpR | AAGGATCCTTACTTGTACAGCTCGTCCATGC |
| mpkA.s | CCGCGGCCGCATGCTCGGGTCCCAATTC |
| mpkA.as | CCCTCGAGGGTCCTGCAAAGCCCTG |
| 5pkcAF | GTGGCCGGGTAGGG |
| pkcA-plR | CACGGAATAGAGACTGC |
| pkcA-plF | GCAGTCTCTATTCCGTG |
| 3pkcA518R-friboB | GTCATCTATCTTCCAACAGGGATTTGACATACTAGAG |
| riboBR | ACTGCAGTCCTGGATAG |
| 3pkcA518F-friboB | CTATCCAGGACTGCAGTAGTGTTGTGTCTCCAAAAG |
| 3pkcA1405R | TGTCGGCTAGATGTGCT |
| 5npkcAF | GTAGGGCCGCACAGG |
| 3pkcA1405R-n | TAGATGTGCTGGAAACTG |
| 5pkcAR-friboB | GTCATCTATCTTCCAACAGGGGTCGTGGTCGGG |
| ALpkcAF-friboB | CTATCCAGGACTGCAGTGAATTCTGAAAAGCTGATTGT |
| pkcA1020R | GATTGAATGTCTCATCCTG |
| pkcA1020R-n | ATCCTGCCACCTGTCGGA |
| pyrG5  pyrG3 | ATGTCTTCGAAGTCCCA  TCAAAGTCCAACTCTTTTCT |
| pyrGRn | CACTTCAGAAGGGAGTATCA |
| mpkA5 | GGGGATCCCTATGCATTATGGACGG |
| 3FLAG-mpkA | AGGATGACGATGTCAAGTCTGACTTACAAGTACAGGGACGG |
| 3xFLAGF | ATGGACTACAAAGACCAT |
| 3xFLAGR | CTTGTCATCGTCATCCT |
| mpkA3 | GGGGATCCGCATGAAACTCTCGGTAAG |
| 5mpkA-FLAG | ATGGTCTTTGTAGTCCATGGCGCGTGAGACTGAGATT |
| 5mpkAF | CTCATTACAGCGATATAGT |
| 5mpkAR-fpyrG | CAGGGATTGCGCCTAGGCCGGGCGCGTGAGAC |
| pyrG-481 | CTAGGCGCAATCCCTGT |
| pyrG-r-new | GCCGGCTTAACCACAG |
| 3mpkAF-fpyrG | CTGTGGTTAAGCCGGCTTAGTCAAGCGTTGGTATT |
| 3mpkAR | ATGTCCAGATCGTCAACT |
| 5mpkAF-n | GCGATATATGGACTATGC |
| 3mpkAR-n | GATGCTAAGCATCCTCG |
|  |  |
