# Supplementary material for: Involvement of Protein Kinase C in the Suppression of Apoptosis and in Polarity Establishment in Aspergillus nidulans under Conditions of Heat Stress
Source: PLoS One. 2012 Nov 28;7(11):e50503. doi: 10.1371/journal.pone.0050503 (PMC3509046; doi:10.1371/journal.pone.0050503)
Supplement: Text S1 — Detailed description of A. nidulans strain constructions. (DOC) [file pone.0050503.s008.doc]

**Text S1. Detailed description of *A. nidulans* strain constructions.**

We constructed the *pkcA*-ts mutants as follows. A 4.4-kb fragment containing the coding region of an N-terminus of PkcA was amplified from the total DNA of the A26 strain using primers, 5pkcAF and pkcA-plR. A 1.0-kb fragment containing the coding region of a C-terminus of PkcA and a downstream of *pkcA* was amplified from the total DNA of the A26 strain using primers pkcA-plF and 3pkcA518R-friboB. A 2.1-kb fragment containing *riboB* was amplified from the total DNA of the A26 strain using primers, riboBF and riboBR. A 0.9-kb fragment containing a downstream of *pkcA* was amplified from the total DNA of the A26 strain using primers, 3pkcA518F-friboB and 3pkcA1405R. These amplified fragments were fused by the fusion PCR using primers, 5npkcAF and 3pkcA1405R-n. The A1145 strain was transformed with the fused fragment. The total DNA of the transformants was extracted as described previously [1] and Southern blot analysis was performed as described previously [2]. The three transformants in which a single copy of the transformed fragment was integrated into the *pkcA* locus confirmed by Southern blot analysis were designated pkcA-ts-2, pkcA-ts-3, and pkcA-ts-5 (Fig. S6A and B).

We constructed strains in which *pkcA* was expressed under the control of *alcA*(p) as follows: A 1.1-kb fragment containing the upstream of *pkcA* was amplified from the total DNA of the A26 strain using primers 5pkcAF and 5pkcAR-friboB. A 2.1-kb *riboB* fragment was amplified from the total DNA of the A26 strain using primers riboBF and riboBR. A 1.4-kb fragment, in which a coding region of the 5’ terminus of *pkcA* was fused to the *alcA*(p), was amplified from pPAALP (Ichinomiya *et al.*, 2007) using primers, ALpkcAF-friboB and pkcA1020R. These amplified fragments were fused by the fusion PCR using primers 5npkcAF and pkcA1020R-n. The A1145 strain was transformed with the fused fragment. The two transformants in which a single copy of the transformed fragment was integrated into the *pkcA* locus confirmed by Southern blot analysis were designated alcA(p)-pkcA-3, and alcA(p)-pkcA-4 (Fig. S6C and D).

We constructed the *bckA* deletion mutants as follows: The A1149 strain was transformed with a 6.2-kb *Hin*dIII-*Spe*I fragment of pbckA::pyroA. The two transformants in which wild-type *bckA* was replaced with a single copy of the *bckA*::*pyroA* fragment at the *bckA* locus confirmed by Southern blot analysis were designated ΔbckA-1 and ΔbckA-2 (Fig. S6E and F).

We constructed the *mpkA* deletion mutants as follows: A 1.2-kb fragment containing the upstream of *mpkA* was amplified from the total DNA of the A26 strain using primers 5mpkAF and 5mpkAR-fpyrG. A 2.0-kb fragment containing *pyrG* was amplified from the total DNA of the A26 strain using primers pyrG-481 and pyrG-r-new. A 1.1-kb fragment containing the downstream of *mpkA* was amplified from the total DNA of the A26 strain using primers 3mpkAF-fpyrG and 3mpkAR. These amplified fragments were fused by the fusion PCR using primers 5mpkAF-n and 3mpkAR-n. The A1149 strain was transformed with the fused fragment. The three transformants in which a single copy of the fragment was integrated into the *mpkA* locus confirmed by Southern blot analysis were designated ΔmpkA-1, ΔmpkA-2 and ΔmpkA-8 (Fig. S6G and H).

We constructed strains in which Lifeact-EGFP was expressed under the control of the *alcA*(p) as follows: The A1149 strain and the pkcA-ts-2 mutant were transformed with a 3.0-kb fragment that was amplified from ppyrGLA using primers pyrG5 and pyrGRn. The two transformants of the wild-type strain, in which a single copy of the amplified fragment was integrated into the *pyrG* locus confirmed by Southern blot analysis, were designated A1149LA-1 and A1149LA-2 (Fig. S5I and J). The two transformants of the *pkcA*-ts mutant, in which a single copy of the amplified fragment was integrated into the *pyrG* locus confirmed by Southern blot analysis, were designated pkcA-tsLA-1 and pkcA-tsLA-2.

We constructed the *bckA* deletion mutants in which Lifeact-EGFP was expressed under the control of the *alcA*(p) as follows: The A1149LA-1 strain was transformed with the 6.2-kb *Hin*dIII-*Spe*I fragment of pbckA::pyroA. The two transformants in which wild-type *bckA* was replaced with a single copy of the *bckA*::*pyroA* fragment at the *bckA* locus confirmed by Southern blot analysis were designated ΔbckALA-1 and ΔbckALA-2.

We constructed strains in which MpkA-FLAG was expressed from its own promoter as follows: A 1.5-kb fragment containing *mpkA* was amplified from pMPKA-pyroA using primers mpkA5 and 3FLAG-mpkA. A 3x*FLAG* fragment was amplified from p3xFLAG-myc-CMV-26 (Sigma) using primers 3xFLAGF and 3xFLAGR. A 4.5-kb fragment containing *pyroA* and the downstream region of *mpkA* was amplified from pMPKA-pyroA using primers mpkA3 and 5mpkA-FLAG. These amplified fragments were fused by the fusion PCR using primers mpkA5 and mpkA3. The A1149 strain and the pkcA-ts-2 mutant were transformed with the fused fragment. The two transformants of the wild-type strain, in which a single copy of the fused fragment was integrated into the *mpkA* locus confirmed by Southern blot analysis, were designated A1149/MF-1 and A1149/MF-2. The two transformants of the *pkcA*-ts mutant, in which a single copy of the fused fragment was integrated into the *mpkA* locus confirmed by Southern blot analysis, were designated pkcA-ts/MF-1 and pkcA-ts/MF-2.

We constructed wild-type strains, which were auxotrophic for pyrimidine as follows: The A1149 strain was transformed with the 2.0-kb *Eco*RV-*Pst*I fragment of ppyrG+750 containing *pyrG* (Yamazaki H., Ohta A., Horiuchi H., unpublished). The three transformants in which a single copy of the fragment was integrated into the *pyrG* locus confirmed by Southern blot analysis were designated A1149/pyrG-1, A1149/pyrG-2 and A1149/pyrG-8.

We constructed the heterokaryons containing both wild-type and *pkcA*-deleted nuclei as follows: A 2.1-kb fragment containing *riboB* was amplified from the total DNA of the A26 strain using primers, riboBF and riboBR. The 1.1-kb upstream and 1.1-kb downstream fragment of the *pkcA* coding region was amplified from the total DNA of the A26 strain using primers, 5pkcAF and 5pkcAR-friboB, and 3pkcAF-friboB and 3pkcAR respectively. These amplified fragments were fused by the fusion PCR using primers, 5npkcAF and 3pkcAR. The A1145 strain was transformed with the fused fragment. The three transformants, which contain both transformed- and untransformed-*pkcA* alleles confirmed by Southern blot analysis, were designated ΔpkcA-h1, ΔpkcA-h2, ΔpkcA-h3.

**References**

1. Oakley CE, Weil CF, Kretz PL, Oakley BR (1987) Cloning of the *riboB* locus of *Aspergillus nidulans.* Gene 53: 293-298.

2. Horiuchi H, Fujiwara M, Yamashita S, Ohta S, Takagi M (1999) Proliferation of intrahyphal hyphae caused by disruption of *csmA*, which encodes a class V chitin synthase with a myosin motor-like domain in *Aspergillus nidulans*. J Bacteriol 181: 3721-3729.
